# Utilising Fourier transform ion cyclotron resonance mass spectrometry (FT-ICR MS) to track the oxidation of lignin by an alkaliphilic laccase†

**DOI:** 10.1039/d4an00124a

**Published:** 2024-03-08

**Authors:** Zak Towle, Faye Cruickshank, C. Logan Mackay, David J. Clarke, Louise E. Horsfall

**Affiliations:** a Institute of Quantitative Biology, Biochemistry and Biotechnology, School of Biological Sciences, University of Edinburgh Roger Land Building King's Buildings Edinburgh EH9 3FF UK towle.zak@gmail.com louise.horsfall@ed.ac.uk; b EaStCHEM School of Chemistry, University of Edinburgh Joseph Black Building David Brewster Road Edinburgh EH9 3FJ UK dave.clarke@ed.ac.uk

## Abstract

Lignin is a complex heteroaromatic polymer which is one of the most abundant and diverse biopolymers on the planet. It comprises approximately one third of all woody plant matter, making it an attractive candidate as an alternative, renewable feedstock to petrochemicals to produce fine chemicals. However, the inherent complexity of lignin makes it difficult to analyse and characterise using common analytical techniques, proving a hindrance to the utilisation of lignin as a green chemical feedstock. Herein we outline the tracking of lignin degradation by an alkaliphilic laccase in a semi-quantitative manner using a combined chemical analysis approach using Fourier transform ion cyclotron resonance mass spectrometry (FT-ICR MS) to characterise shifts in chemical diversity and relative abundance of ions, and NMR to highlight changes in the structure of lignin. Specifically, an alkaliphilic laccase was used to degrade an industrially relevant lignin, with compounds such as syringaresinol being almost wholly removed (95%) after 24 hours of treatment. Structural analyses reinforced these findings, indicating a >50% loss of NMR signal relating to β-β linkages, of which syringaresinol is representative. Ultimately, this work underlines a combined analytical approach that can be used to gain a broader semi-quantitative understanding of the enzymatic activity of laccases within a complex, non-model mixture.

## Abstract 2

Herein, we outline a method to semi-quantitatively analyse and track the enzymatic oxidation of lignin using high resolution mass spectrometry.

## Graphical abstract

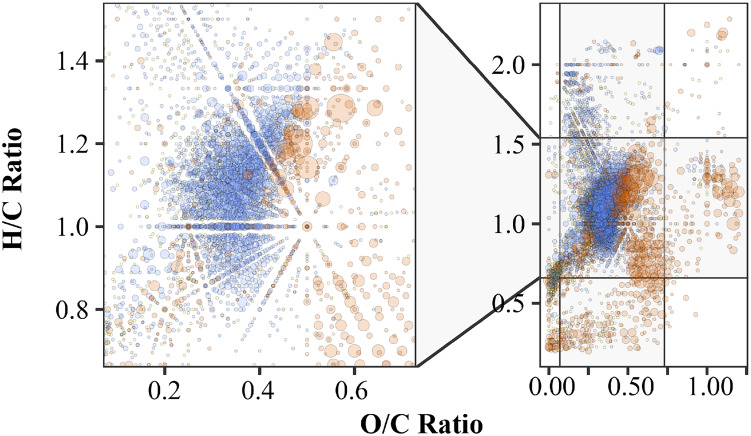

## Introduction

Lignin is one of the most abundant biopolymers on Earth, comprising approximately 30% of plant secondary cell walls and has the potential to replace fossil fuels as a feedstock to produce fine chemicals due to its aromatic structure.^1^ Its main function in plants is to provide structural rigidity and vascularity whilst acting as a protective barrier against pathogens. Currently, it is a major by-product of the paper-pulp and biofuel industries and is usually burnt in recovery boilers to fuel respective processes. The aromatic structure of lignin is the product of radical crosslinking of phenylpropanoids, syringyl (S), guaiacyl (G) and *p*-hydroxyphenyl (H) units, derived from sinapyl, coniferyl and *p*-coumaryl alcohols, leading to an inherently complex structure containing an array of recalcitrant linkages and bonds.^2,3^ The ratio of each phenylpropanoid unit can vary significantly between species of plant, with softwood lignins usually being comprised of G units with a small amount of H units, whilst hardwood lignins are often comprised of G units and S units in varying numbers. These units are linked by β-O-4, β-5, α-O-4, 5-5, 4-O-5, β-1 and β-β linkages, where β-O-4 units are the most dominant in both hardwood and softwood lignins representing approximately 60% and 45–50% respectively and are also the most amenable to degradation. The recalcitrance of lignin provided by this array of different linkages has proved a hindrance for the utilisation of lignin as a green chemical feedstock; with a more comprehensive understanding of lignin and its depolymerisation required for these processes to succeed. Currently, commercial uses of lignin are restricted to binding agents and additives, with a few applications for fine chemicals other than vanillin.^4–7^ However, there have been numerous strategies employed to effectively depolymerise lignin utilising enzymes as green catalysts,^8–12^ where successful enzymatic approaches pose a low energy, non-toxic and green alternative to common pyrolytic techniques.

One such class of enzymes, laccases (EC 1.10.3.2) have shown promise in the enzymatic depolymerisation of lignin.^10,13–15^ Laccases are enzymes produced by a wide range of organisms including bacteria, fungi, and plants where they are implicated in the synthesis of lignin in plants and depolymerisation of lignin in bacteria^16,17^ and fungi.^18,19^ Laccases have been further utilised in the bioremediation of industrial wastes such as phenolic dyes, and as biobleaching agents.^19–23^ However, laccases often have a relatively low redox potential when compared to other ligninolytic enzymes and therefore require small mediators to oxidise non-phenolic substrates.^24–29^ Moreover, laccases often have optimal activities in acidic conditions,^18^ making their application to lignin depolymerisation difficult due to lignins’ inherent insolubility in aqueous systems at low pH. The alkaliphilic laccase used herein is an engineered bacterial laccase (MetZyme) of industrial relevance that has previously been implicated in both the depolymerisation of lignin for chemical synthesis^30^ and polymerisation for coating applications.^31^

The complexity of lignin mixtures, both structural and chemical, makes it difficult to analyse when compared to more ordered polymers and although nuclear magnetic resonance (NMR) spectroscopy, Fourier transform infrared (FT-IR) spectroscopy and gel permeation chromatography (GPC) approaches can elucidate linkages,^32,33^ structural changes^9,34,35^ and deconvolute polymer characteristics,^36,37^ fully understanding the chemical diversity of lignin is a more challenging task. The type of analysis chosen should also consider the extraction method used to isolate the lignin from lignocellulose. For example, lignins’ derived from the Kraft process, which includes harsh physiochemical treatments, contain a much higher chemical diversity of compounds due to both degradation and repolymerisation.^38^ Conversely, the extraction of lignin using organosolv methods yield lignin more akin to lignin's natural form^39,40^ and therefore a less complex chemical diversity which is more readily amenable to a range of analytical methods. Within this study we use a lignin derived from the liquid hot water pre-treatment and enzymatic bioprocessing of *Betula pendula*,^41–43^ European white birch, with the chemical diversity arising from this lignin extraction method still a relative unknown. Although, it has been highlighted that LHW processing can result in the cleavage of β-O-4 ether linkages which can in turn transform into enol-ether compounds or repolymerise into other structures.^44–46^

To analyse the chemical diversity of lignin effectively, several mass spectrometry strategies have been employed, utilising different ionisation methods, which again can greatly impact the subsequent analysis of lignin. More gentle ionisation techniques such as electrospray ionisation (ESI) and atmospheric photoionisation (APPI) and atmospheric pressure chemical ionisation being among the most used ionisation methods to reduce the fragmentation of lignin compounds during ionisation.^47–51^ Whereas matrix-assisted laser desorption ionisation (MALDI) methods have also been used to study lignin to varying degrees of success with a recent study showing improved detection using graphite-assisted laser desorption ionisation of insoluble and soluble (Lignoboost & Kraft) lignin compounds.^52^ A comprehensive overview of different MS methods and how they can be applied to lignin samples is outlined by Letourneau and Volmer.^53^ It has been shown that low-resolution MS methods (including LC-MS approaches) can be readily implemented for targeted analyses of specific compounds, including of bio-oils.^54^ However, untargeted approaches for profiling lignin's chemical complexity require the use of high resolution or ultrahigh resolution MS methods (HRMS/UHRMS) and, in particular, Fourier transform ion cyclotron resonance (FT-ICR) MS shows great promise in this area.

Unlike low-resolution methods, FT-ICR MS enables the detection of thousands of ions concurrently with a very high mass resolution and accuracy, allowing for the assignment of elemental formulae of individual compounds in extremely diverse and complex mixtures. FT-ICR MS has shown great promise in the semi-quantitative analysis of complex chemical mixtures including dissolved organic matter (DOM),^55–57^ oil products,^58–61^ whisky^62,63^ and lignin.^50,64–67^ To date most HRMS studies of lignin have focused on method development,^47,68–70^ broad analysis of lignin samples,^29,67,71,72^ the physiochemical degradation of lignin^65,66,70,73^ or the analysis of small model compounds which are unrepresentative of complex industrial lignins.^28,74^ FT-ICR MS has been applied in only a handful of lignin degradation studies often analysing the fungal degradation of lignin or wood.^64,72,75,76^ This includes a study which identified a range of phenolic compounds arising from the decomposition of wood by a brown-rot fungus, *Rhodonia placenta*, using MALDI-FT-ICR MS.^64^ Recently, we reported the first study using FT-ICR MS to examine the ligninolytic activity of a laccase from *T. versicolor* on Kraft lignin.^50^ Herein we build upon this previous study and highlight the ability of high-resolution MS to monitor the global changes in the composition of a high-molecular weight industrial lignin (*M*_w_ > 70 kDa) after laccase treatment and the fluctuations in abundances of individual ions. Moreover, using complementary 2-D HSQC NMR analysis, we highlight specific laccase action on β-β linkages.

## Results and discussion

### FT-ICR MS analysis

**Fig. 1:**
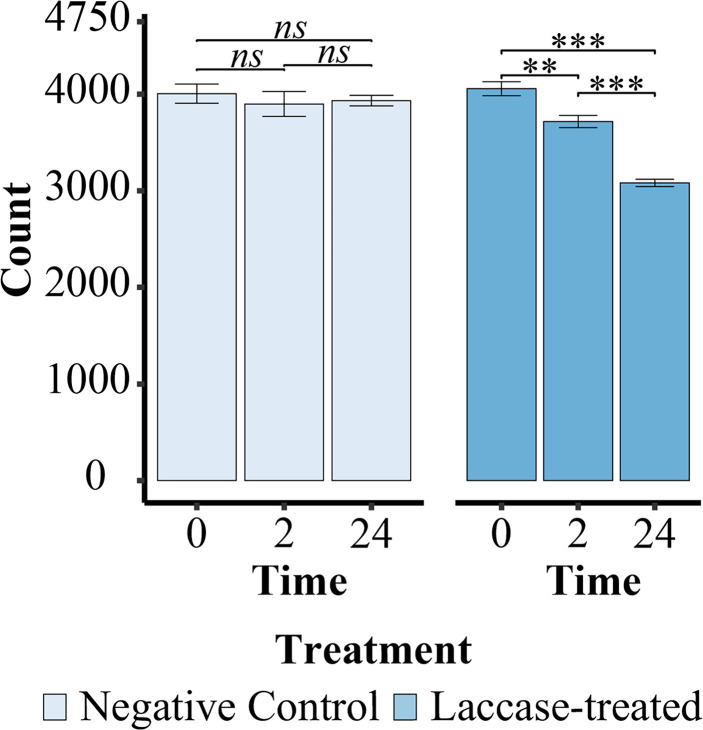
Average number of ions assigned in the negative control and laccase-treated sample. There is a significant decrease in the number of compounds detected in the laccase-treated sample over time, ANOVA *p* < 0.001 ‘***’ and *p* < 0.01 ‘**’. Whereas there is no significant variation observed in the negative control sample.

In total, 18 spectra were obtained pertaining to 0-hour, 2-hour and 24-hour time points of the incubation of METNIN™ Macro lignin with the alkaliphilic MetZyme® laccase. After data curation 26 731 ions were assigned across the 18 spectra, with average compositions and the unique ions assigned for each treatment and timepoint displayed in Table 1. All ions were assigned using Formularity^77^ and only included ions comprised of C, H, O and S, with all ^13^C-containing ions removed. After data processing and ion assignment the average number of assigned ions in the undigested lignin samples was consistent with previous lignin FT-ICR MS studies, with an average of 4005 (negative control) and 4057 (laccase-treated) ions at time zero, Fig. 1, compared to 3350,^50^ 1199–2793,^73^ 4201 ^47^ & 4740–6600.^78^ As FT-ICR MS does not distinguish between isomers of the same *m*/*z*, multiple different candidate compounds may exist for each distinct *m*/*z*.

**Table 1:** Summary of characteristics identified by FT-ICR MS analysis of degraded (laccase-treated) and non-degraded (negative control) lignin. Standard deviation is shown in brackets and all ^13^C-containing ions are excluded

Treatment	Timepoint (hours)	Average no. of assigned ions	Double bond equivalents (DBE)	C	H	O	*M*_w_ (Da)	O/C ratio	H/C ratio
Negative	0	4005	15.4 (10.2)	32.3 (14.3)	35.7 (16.9)	10.5 (5.4)	606.4 (229.0)	0.37 (0.22)	1.15 (0.37)
	2	3916	15.3 (10.0)	31.3 (13.8)	34.0 (15.7)	10.5 (5.3)	593.6 (220.0)	0.38 (0.22)	1.13 (0.37)
	24	3933	15.4 (10.2)	31.7 (14.1)	34.5 (16.7)	10.6 (5.4)	599.2 (225.5)	0.38 (0.22)	1.14 (0.38)
Laccase-treated	0	4057	14.9 (10.1)	31.6 (14.1)	35.3 (17.2)	10.5 (5.4)	597.2 (226.0)	0.38 (0.22)	1.16 (0.38)
	2	3716	14.7 (9.5)	30.2 (13.3)	33.0 (16.5)	10.5 (5.4)	579.6 (215.3)	0.39 (0.23)	1.13 (0.38)
	24	3081	13.7 (9.7)	27.5 (11.7)	29.5 (15.5)	10.2 (5.3)	538.6 (191.7)	0.42 (0.24)	1.13 (0.42)

**Fig. 2:**
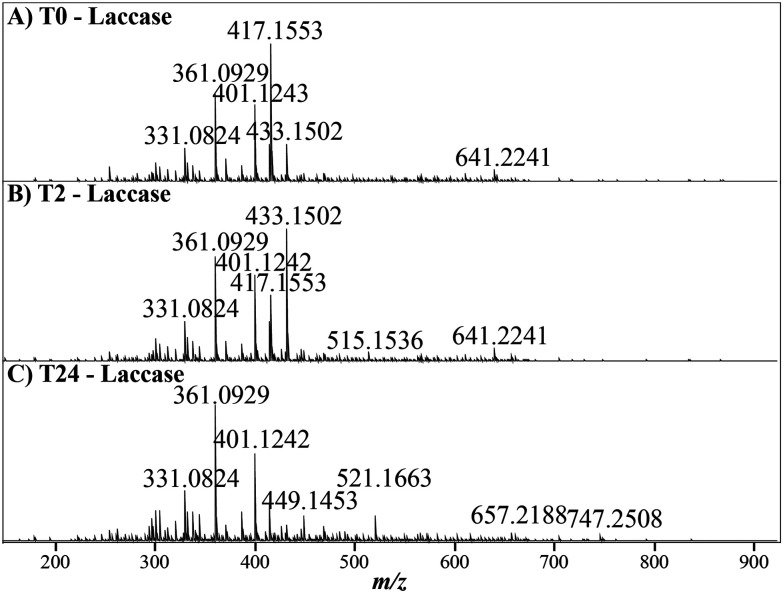
The mass spectrum of a laccase treated METNIN™ lignin sample at 0 hours (A), 2 hours (B) and 24 hours (C), with the most abundant ion in each spectra representing 100% relative intensity. There are already noticeable compositional changes, with the most abundant ion at 417 *m*/*z* steadily declining across the time course and the ion at 433 *m*/*z* increasing after 2 hours before declining again after 24 hours.

Within the untreated lignin there was no change in the number of ions assigned over the time course (Fig. 1). However, the mass spectra across the laccase treatment time course display noticeable shifts in intensity and chemical diversity, particularly for abundant ions at 417, 433, 449 and 521 *m*/*z*, Fig. 2, and there was a significant reduction of the number of assigned ions after both 2 and 24 hours (ANOVA, T2 = *p* < 0.01, T24 = *p* < 0.001, Tables S2 & S5†), with ∼1000 ions lost over the time course, Fig. 1. Moreover, there is minimal assignment bias in both treatments with only a 7% reduction in the number of assigned ions after 24 hours in the laccase-treated samples compared to the loss of 24% of the ions originally assigned at time 0, with no reduction in overall assignment or loss of ions observed in the negative control, Fig. 1 & Table S1.†

**Fig. 3:**
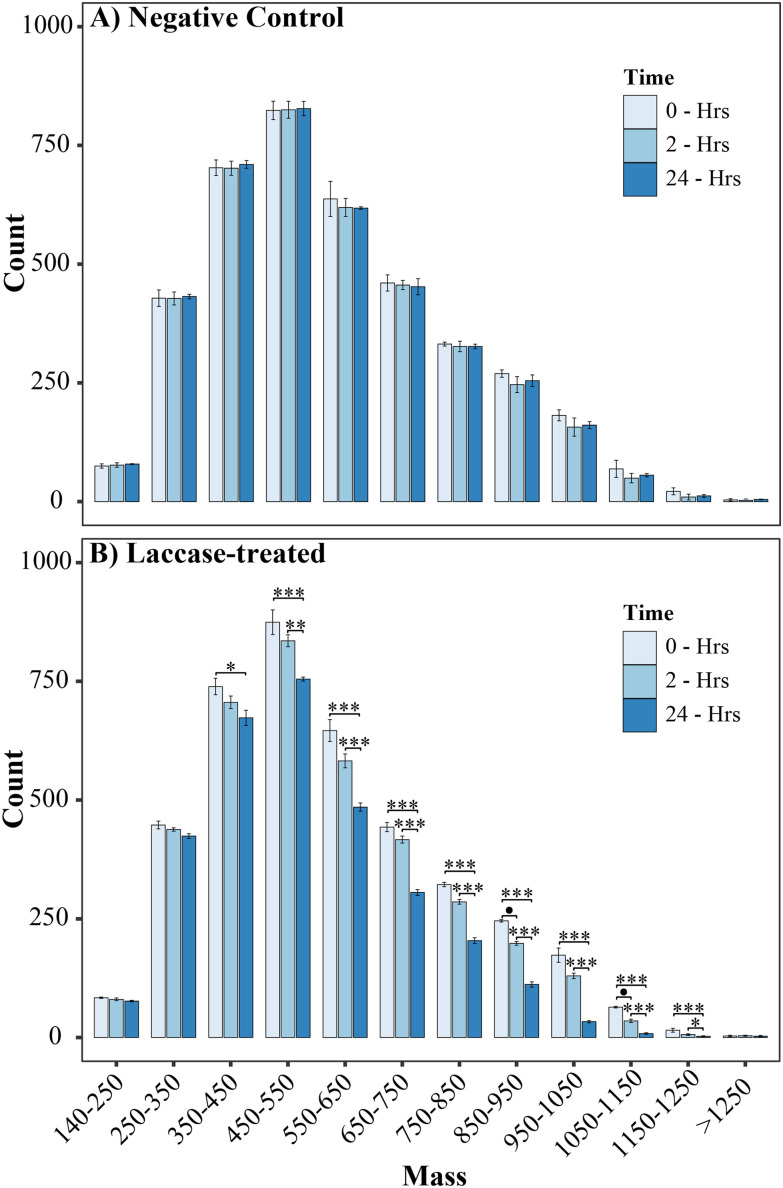
Mass distribution of compounds assigned in the (A) negative control and (B) laccase-treated sample, across timepoints. Error bars represent the standard deviation of count for biological replicates. Significant differences in peak number between treatment and time across each mass bracket were calculated using a Poisson GLM; *n* = 3, *p* < 0.1 ‘●’, <0.05 ‘*’, <0.01 ‘**’, <0.001 ‘***’.

Therefore, laccase treatment reduces the observable chemical diversity of the lignin, as reported previously,^50^ and could correspond to enzymatic activity on larger lignin structures, with the resulting ions falling into pre-existing *m*/*z* values already present at 0-hours. However, it could also be the case that smaller compounds are also being modified by the laccase and detected at different *m*/*z* values. Complex mixture profiling by FT-ICR MS is limited by the *m*/*z* range of what can be accurately profiled. Consequently, small phenolic monomers may go undetected. This drop in ion assignment in the laccase treated lignin was further assessed by binning ions according to their assigned mass, allowing the detection of significant reductions in count over the time course, Fig. 3. The decrease in number of assigned ions occurred most considerably within the higher mass ranges, for example, reductions of 37%, 55% and 80% were observed for 750–850 Da, 850–950 Da and 950–1050 Da respectively after 24 hours (Poisson GLM, *p* < 0.001, Tables S6 & S7†). This suggests that the alkaliphilic laccase targets larger compounds, with the absence of increases in count above 800 Da signifying that under these conditions the laccase is acting to depolymerise the lignin.

### Heteroatomic class analysis

**Fig. 4:**
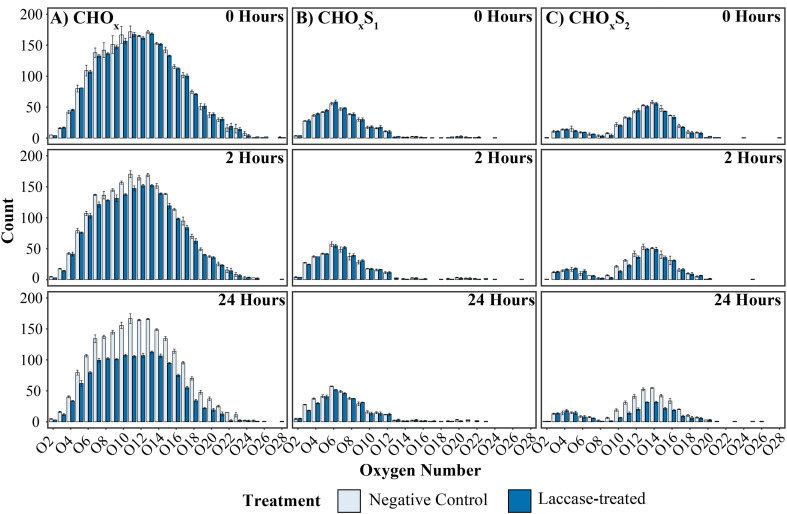
Histogram of heteroatomic classes of lignin-like compounds in the negative control and laccase treated samples. Panels (A, B and C) represent CHO_*x*_, CHO_*x*_S_1_ and CHO_*x*_S_2_ ions with time points shown from top to bottom. There are significant falls in the number of CHO_*x*_ and CHO_*x*_S_2_ compounds after 24 hours of laccase treatment and no noticeable changes in the number of CHO_*x*_S_1_ compounds. Error bars represent the standard deviation of the number of ions in each heteroatomic class.

To further investigate the activity of the alkaliphilic laccase, heteroatomic class analyses of the lignin-like ions was conducted, Fig. 4. As expected, at 0-hours both treatments were similar in distribution. However, following 2 hours of treatment, noticeable decreases within the CHO_*x*_ and CHO_*x*_S_2_ classes were identified. After 24 hours, substantially larger reductions in the number of ions identified in these classes were noted, with significant decreases of 31%, 36%, 35%, 32% and 28% observed for CHO_10–14_ respectively (Poisson GLM, *p* < 0.01 – *p* < 0.001, Table S8†). Significant reductions were also detected for CHO_12–15_S_2_ with reductions of 45%, 33%, 36% and 36% respectively (Poisson GLM, *p* < 0.01 – *p* < 0.001, Table S9†). Conversely, the CHO_*x*_S_1_ compounds remained comparable across time and treatments, suggesting that compounds containing multiple sulfur groups are more readily accessible to enzymatic oxidation than mono-sulfur compound classes.

### van Krevelen analysis

**Fig. 5:**
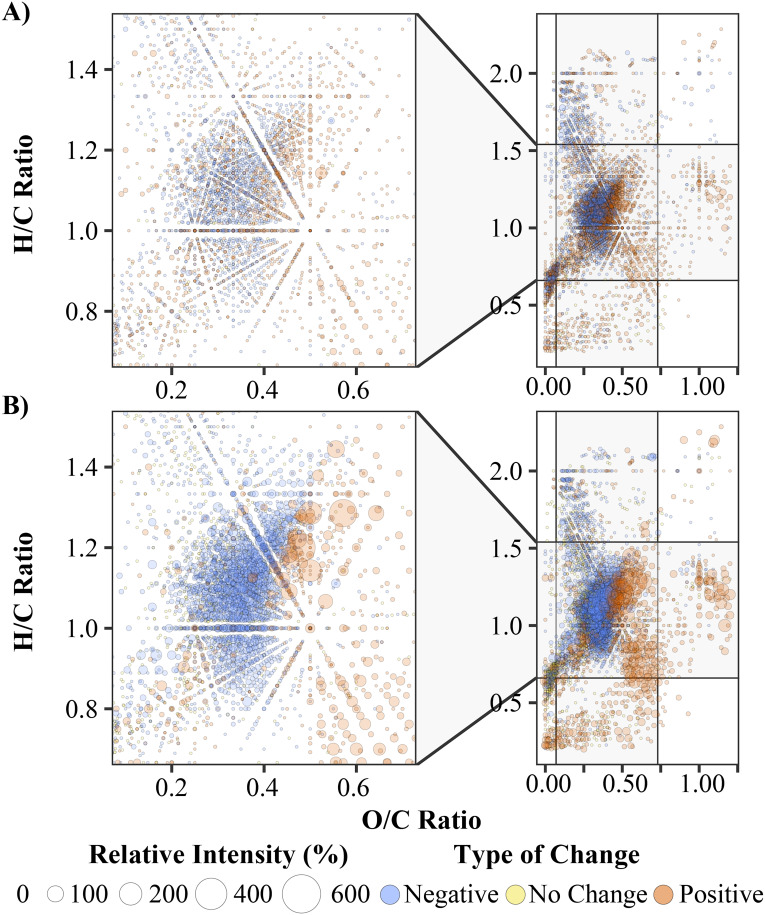
van Krevelen diagrams of the undigested negative control lignin (A) and the MetZyme® laccase depolymerized lignin (B) after 24 hours. The whole plot is shown on the right-hand side, with the lignin-like region magnified on the left-hand side. There are minimal changes in relative intensity in the negative control, within the lignin-like region, whereas there are numerous changes of >100% RI which occur after 24 hours of laccase treatment. Notably, there is a pattern of lower O/C ratio ions decreasing in RI and higher O/C ratio compounds increasing in RI after laccase treatment.

To assess each ion based on their abundance, the data were analysed using van Krevelen diagrams^79^ (VKD), which separate ions based on their hydrogen to carbon (H/C) and oxygen to carbon ratios (O/C). These plots allow for the more quantitative analysis of thousands of ions simultaneously and further allow the broad classification of ions. Initially VKDs used relative intensity (RI) and identified minimal shifts in H/C and O/C ratios, Fig. S3,† with the most notable reductions occurring after 24 hours of laccase treatment between H/C = 0.30–0.43 and O/C = 1.15–1.30. However, when calculating the percentage change in relative intensity across timepoints for each ion, these shifts become clearer and allow for improved semi-quantification. Each ion was also filtered to remove any ion that did not change by ±20% absolute intensity over the time course, removing ions that changed significantly in relative intensity, but which had no shift in absolute intensity. This resulted in the removal of 1095 ions, and subsequent tracking of 4569 distinct ions over the time course. Of these 4569 ions, 3118 were classified as ‘lignin-like’, with VKD of these ions and their percentage change in RI after 24 hours shown in Fig. 5. Notably, most ions at lower O/C values (0.2–0.4) decrease in RI, with ions at higher O/C values (0.4–0.7) broadly increasing in RI. This oxidation pattern was also observed in the negative control, likely due to background oxidation of the lignin, although the degree of change in RI is considerably lower when compared to the ions in the laccase-treated sample.

### Semi-quantitative analyses

**Fig. 6:**
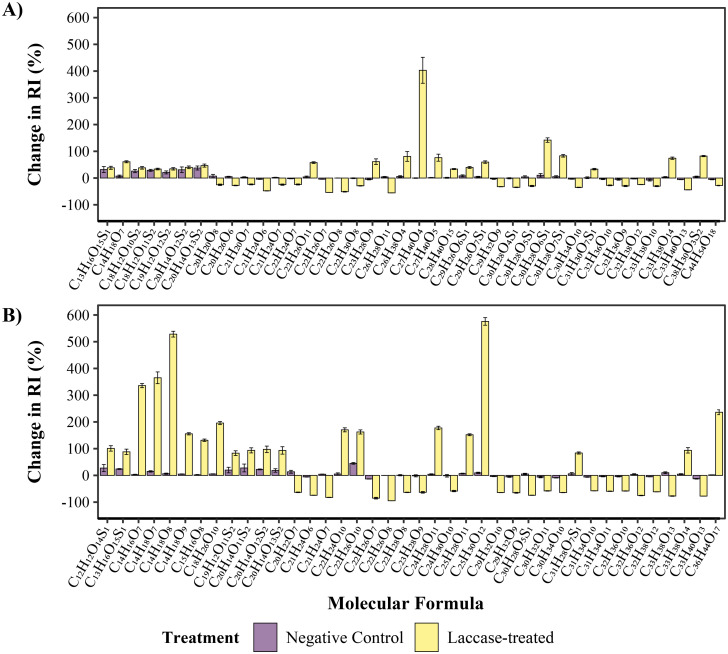
Change in intensity of compounds after (A) 2 hours and (B) 24 hours after treatment with Metgen laccase (molecular formulae and not ionic formulae is shown). The top 40 compounds that increased or decreased the most in intensity after laccase treatment are highlighted. (A) Substantial shifts can be seen after 2 hours of laccase treatment with the highest increase in intensity after 2 hours being C_27_H_40_O_4_ with an increase of 403% ± 49 and largest decrease being C_26_H_28_O_11_ with a change in intensity of 55.5% ± 0.2, with minimal changes observed in the negative control. (B) After 24 hours there are even more large shifts in intensity, with the highest increases now being 575% ± 14 and 528% ± 11 for C_25_H_30_O_12_ and C_14_H_17_O_8_, respectively. The largest decrease in intensity is a 94.8% ± 0.1 drop in signal of C_22_H_26_O_8_, the compound with the highest RI at 0 hours. Error bars indicate the standard deviation of percentage change in RI of ions between each timepoint, with some compounds that decrease substantially lacking error bars due to the loss of signal in at least one of the three biological replicates.

Individual molecular formulae identified and assigned using FT-ICR MS were also investigated, Fig. 6. After 2 hours the largest increase in RI within the laccase-treated sample was 403.0% ± 48.5 (−0.2 ± 1.5: NegCtrl) for C_27_H_40_O_4_ and a decrease of −55.5% ± 0.2 (4.2 ± 1.7: NegCtrl) for C_26_H_28_O_11_. Additionally, after 24 hours the largest shifts in RI correspond to −94.8 ± 0.1 for C_22_H_26_O_8_ (0.0% ± 0.0: NegCtrl) and an increase of 575.8% ± 14.2 (9.5% ± 2.5: NegCtrl) for C_25_H_30_O_12_. In this case, C_22_H_26_O_8_ corresponds to syringaresinol (confirmed *via* MS fragmentation experiments, Fig. 8), with other resinol-related formulae such as C_22_H_26_O_7_ also decreasing substantially in RI after laccase treatment (−53.8% ± 0.2 hours and −85.1 ± 3.1 after 24 hours). This indicates that these resinol compounds and their β-β linkages are targeted and modified by the laccase. Although they may not be wholly degraded, phenol oxidising enzymes such as laccases, have been implicated in the oxidation and destruction of β-β linkages,^29,80,81^ with this activity previously identified for the MetZyme® laccase.^31^ There were numerous other ions that increased substantially in RI due to laccase activity, including potential phenyl glucosides, C_14_H_18_O_8_ (+528%), C_14_H_18_O_7_ (+364%), C_14_H_18_O_9_ (155%), C_15_H_16_O_8_ (131%) and other potential oxidised resinol structures; C_22_H_24_O_10_ (+170%), C_22_H_26_O_10_ (+162%) and C_24_H_28_O_11_ (+177%). Although FT-ICR MS is semiquantitative, these changes are starkly different from the negative control, suggesting a large shift in the abundance of these ions.

### 2-D ^1^H–^13^C HSQC NMR

**Fig. 7:**
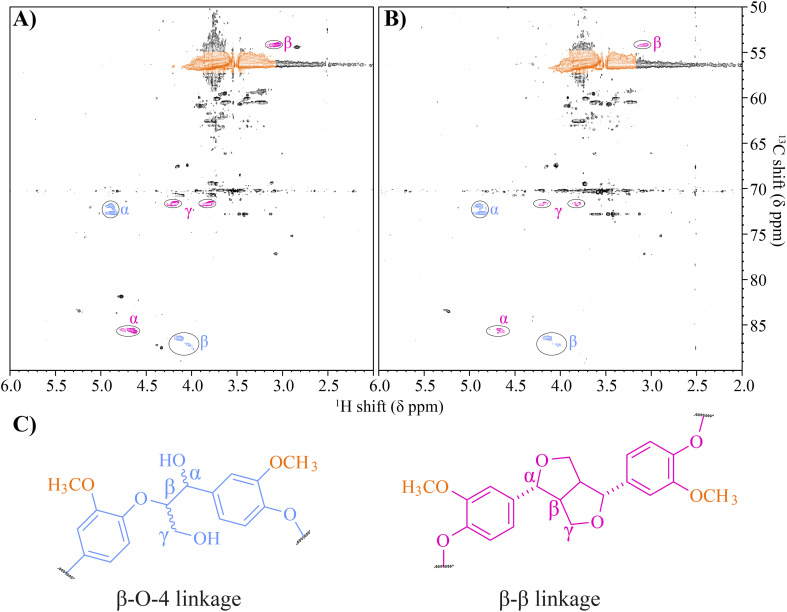
NMR spectra of the (A) negative control and (B) the laccase treated Metgen fraction 3 lignin. Peaks corresponding to protons within different linkages have been circled, with their corresponding location in their respective linkages highlighted with letters and shown in panel (C). The peaks are coloured according to each linkage, with methoxyl peaks shaded in orange, β-O-4 linkages in blue and β-β linkages in pink. Integral values for the γ1, γ2, α and β protons in the β-β linkage, pink, for the laccase treated lignin were lower by 64.0% ± 10.0, 44.7% ± 8.7, 50.0% ± 10.6 and 48.7% ± 2.1 respectively compared to the negative control. However, integral values for the α and β protons for β-O-4 linkages fell by only 0.848% ± 6.2 and 3.23% ± 7.0 compared to the negative control.

2-D HSQC NMR has been widely used across many different lignin types for the quantification of specific lignin linkages and to gauge the level of depolymerisation.^9,29,35,73,82–84^ NMR analysis estimated the untreated β-O-4 and β-β linkage content to be relatively low at 4.3 and 1.7 per 100 C9 units, respectively, likely due to the lignin not being acetylated prior to analysis. To further confirm that β-β linkages were targeted by the laccase in this study, NMR analysis was performed on the 24-hour treated samples. In the laccase treated lignin, large decreases in integral values of 46–62% were observed in the proton environments, indicative of β-β linkages, specifically the α, β & γ protons at *δ*_C_/*δ*_H_ 71.6/4.2 (γ1), 71.7/3.8 (γ2), 85.6/4.7 (α) and 3.1/54.0 (β), Fig. 7 & Table S10.† There were no notable changes in the intensity of peaks associated with β-O-4 linkages (0.96% ± 10 and 0.07% ± 8), perhaps owing to the lack of a mediator compound in the digestion system preventing oxidative attack of non-phenolic aromatics. The NMR experiment was also unable to detect the signal corresponding to the γ proton of the β-O-4 unit or any signal corresponding to β-5 linkages, which were flooded out due to the high noise presence of an industrial PEG-like contaminant present at 3.51 ppm ^1^H.

### FT-ICR MS CID fragmentation

**Fig. 8:**
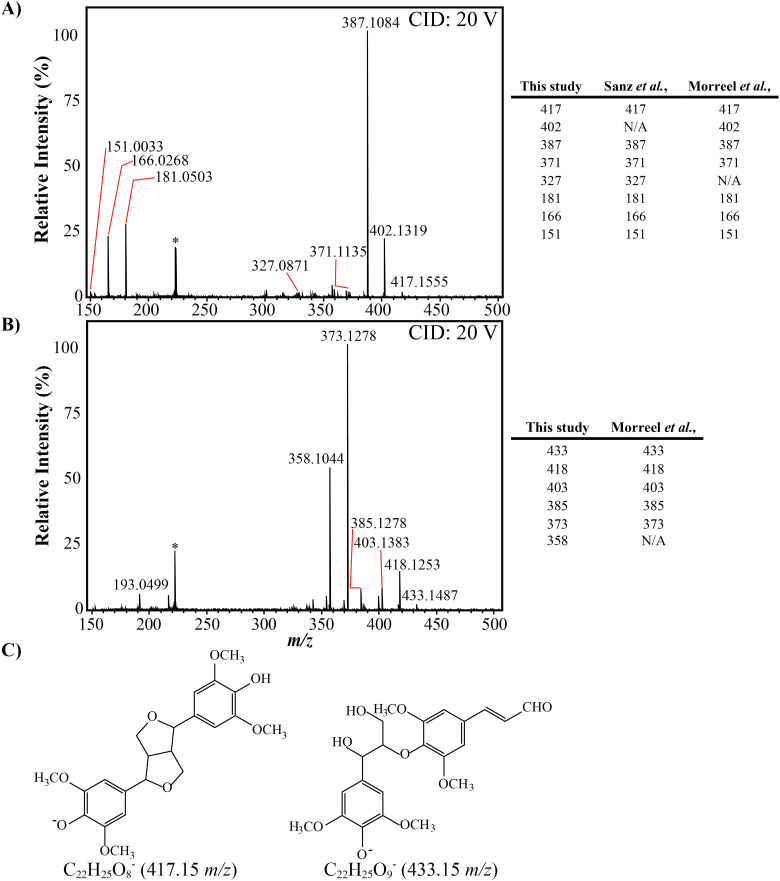
Collision induced dissociation mass spectra of ions present at 417 *m*/*z* (A) and 433 *m*/*z* (B), with the highest peak in each spectra representing 100% relative intensity. Peaks of interest are highlighted in the side panels of (A) and (B) with comparisons to MS/MS studies of the candidate compounds.^85,87^ The potential structures of the candidate ions are highlighted in panel (C). A contaminating peak at 233 *m*/*z*, representing background noise, is labelled with an asterisk (*).

The most abundant ion after 2 hours of laccase treatment, 433.1502 *m*/*z* (C_22_H_25_O_9_ [M − H]^−^), was left unassigned by Formularity. Initially, as this ion differed from the most abundant ion at time zero, (C_22_H_25_O_8_ [M − H]^−^, 417.1554 *m*/*z*), by the accurate mass of oxygen, it was thought that it could represent a more oxidised form of C_22_H_25_O_9_ [M − H]^−^, potentially representing syringaresinol (S-β-β-S) and was investigated further. Subsequent collision induced dissociation (CID) tandem MS experiments were employed for structural characteristics of both ions, Fig. 8. The fragmentation pattern for the ion at 417 *m*/*z*, which decreased substantially over the time course, aligned well with previous literature values for syringaresinol (S-β-β-S), Fig. 8A.^85–87^ The fragmentation pattern of the ion at 433 *m*/*z* also agreed well with previous studies of the syringyl dimer in a β-O-4 conformation (S-β-O-4-S), Fig. 8B,^85^ highlighting that the candidate ion likely did not represent an oxidised form of syringaresinol as initially thought and instead could be a S-β-O-4-S structure. The potential structures of both candidate ions is shown in Fig. 8C. Interestingly, the ion at 433 *m*/*z* varied considerably in RI across the time course, increasing from 29% RI (intensity: 1.1 × 10^9^) to 100% RI after 2 hours (intensity: 3.2 × 10^9^), before declining to only 10% RI after 24 hours (intensity: 7.0 × 10^6^). As NMR analyses indicated there was no decrease in signals corresponding to β-O-4 linkages there is potential that the ion at 433 *m*/*z* was released from a larger lignin oligomer before being further modified by the laccase without destruction of the β-O-4 linkage, or changes in the associated α and β proton environments.

## Conclusions

In conclusion, this work demonstrates the capability of FT-ICR MS analyses to track the oxidative activity of laccases on lignin in a semi-quantitative manner, highlighting the significant loss of compounds due to enzyme activity across different mass ranges and heteroatomic classes. ESI-FT-ICR MS identified syringaresinol as being targeted by the MetZyme® laccase (95% decrease), with this being reaffirmed by the β-β cleavage detected by HSQC NMR (>50%). Moreover, tandem MS methods were utilised to structurally identify individual ions with specific chemical formulae; in this case confirming the assignment of syringaresinol and a syringyl–syringyl β-O-4 with fluctuating relative ion abundance. However, little to no oxidative cleavage of the more prevalent β-O-4 linkages was indicated by NMR, likely due to the absence of a mediator system preventing oxidation of non-phenolic compounds. Although laccases are often deemed as randomly acting enzymes, these results demonstrate that they can be utilised in a more targeted approach to degrade specific lignin linkages. This work exemplifies how FT-ICR MS can be employed for the rapid analysis of lignin mixtures, helping to develop and optimise degradation systems through the identification of target compounds and linkages, resulting in more streamlined and focused processes. These FT-ICR MS analysis methods could further by employed to follow the activity of any enzyme in a complex mixture in a semi-quantitative manner, assessing both changes in chemical diversity as well as highlighting ions of particular interest.

## Experimental

### Enzymatic digestion

The lignin used in this study is the industrially produced METNIN™ Macro, a lignin derived from the LHW treatment and enzymatic hydrolysis of *Betula pendula* (European white birch, hardwood), from the SWEETWOODS project and was supplied by Metgen Oy. The specific METNIN™ Macro fraction is representative of the crude lignin soluble in alkaline environments for Metgens’ subsequent fractionation process. The alkaliphilic laccase utilised was a commercially available, genetically evolved laccase of bacterial origin, MetZyme® LIGNO™ and was also supplied by Metgen Oy. 20 mM ammonium acetate pH 9.5 was made by first creating a 200 mM ammonium acetate solution (Fisher Scientific) with drop-wise additions of ammonium hydroxide (30% Sigma Aldrich) to bring the solution to pH 9.5 using a pH probe (Mettler-Toledo). The laccase was filter sterilised using a 0.22 μm Stericup® (Merck Millipore) and activity determined against 2,6-dimethoxyphenol (2,6-DMP, Sigma Aldrich) as an oxidation substrate in 20 mM ammonium acetate pH 9.5. To prevent contamination of the experimental samples an additional flask comprised of lignin in 20 mM ammonium acetate pH 9.5 was used to confirm that were no background fluctuations in pH across the time course. Digestion reactions were setup according to Table 2, with a total volume of 30 mL and were continuously shaken at 200 rpm and 25 °C for 24 hours, in 100 mL flasks (heat treated at 450 °C for 8 hours to remove any organic material). The digestion reaction was setup in triplicate, alongside three undigested (no laccase) negative controls. The reaction mix was sampled at three time points, 0 hours, 2 hours and 24 hours, with 2 × 1 mL aliquots taken at each point and diluted 1 : 1 in Chromasolv™ methanol (Honeywell) to stop the reaction prior to storage at −80 °C. After 24 hours, the remaining reaction mixture was frozen in liquid nitrogen and freeze dried.

**Table 2:** Reaction conditions for lignin digestion experiments

Reagent	Final concentration
MetGen laccase	3.1 U mL^−1^ a
200 mM ammonium acetate pH 9.5	20 mM
Lignin (METNIN™ Macro Soluble)	2.5 g L^−1^

a U mL^−1^ references the U L^−1^ of laccase per mL of reaction mixture (a total of 93 U was added to the reaction). This activity was chosen corresponds to the same activity used in previous work.^50^

### Sample preparation

100 μL of each sample was diluted 1 : 10 in 50% methanol : water (Honeywell, Chromasolv™) and analysed spectrophotometrically at 280 nm in a 96-well UVStar® plate (Greiner Bio-One) and compared to a standard curve of Sigma Kraft Lignin (Sigma Aldrich, 471003), Fig. S1,† to determine the overall aromatic content. Concentrated samples were filtered using a 0.2 μm PTFE filter (VWR) and diluted in 50% methanol : water to obtain a final concentration of 150–175 μg mL^−1^ lignin.

### ESI-FT ICR MS analysis

The mass spectra were acquired on a 12 T Bruker SolariX 2XR FT-ICR MS (Bruker Daltonics, Germany) with an electrospray ionisation (ESI) source in negative mode, with nebuliser gas flow set to 1.0 bar with drying gas at 4 L min^−1^ at 200 °C. Spectra were acquired using 300 summed scans between 147.43 *m*/*z* and 3000 *m*/*z* into a 4 MW FID of 1.677 s. Time of flight was set to 0.800 ms with an accumulation time of 100 ms. The three replicates of each treatment at time points 0, 2 and 24 hours, associated ‘enzyme-only’ blank control samples, and three quality control (QC) replicates were analysed over two days. QC samples were run at specific time points across the course of the analysis to ensure reproducibility. Samples were then applied to the system at a flow rate of 120 μL h^−1^, with the syringe and line being purged with 50% methanol and air two times between sample loadings.

### Fragmentation analysis

Lignin samples were also subject to fragmentation studies. Ions at 417 and 433 *m*/*z* were isolated using the quadrupole and a 2 *m*/*z* window, with an accumulation time of 5 seconds, a time of flight of 0.700 ms across 25 scans, in a 4 M file, on the same FT-ICR MS as previous experiments. Collision induced dissociation (CID) was utilised to fragment the ions with collision voltages of 5, 10, 15 and 20 V, with spectra collected as above. Where required, individual *m*/*z* ions were isolated using a combination of the quadrupole and sustained off resonance irradiation (SORI) in the ICR cell before CID fragmentation. Spectra for the CID experiments alongside mass lists can be found in Fig. S8–S11 and Tables S11, S12.†

### Quality control analysis

The mass spectra were left uncalibrated prior to assignment in Formularity (Pacific Northwest National Laboratory, U.S. DOE). Peaks that were identified in multiple replicates were processed to calculate the coefficient of variation to ensure replicability of the instrument, Fig. S2.†

### Calibration list creation

Isotopic fine structure analysis was conducted for high intensity peaks to determine each chemical formula, with high-certainty predictions being included in the calibration list. The spectra also contained traces of polymers at >400 *m*/*z* that increased by C_2_H_4_O, including C_32_H_57_O_11_ at 617.38894 *m*/*z*, which allowed them to be easily identified and used in the calibration list. The spectra were iteratively calibrated with known compounds to help ease the identification across the *m*/*z* range. In total, 63 distinct ionic formulae were confirmed and used to calibrate the experimental broadband mass spectra using a quadratic function, Table S1.† All peaks which conformed to the quadratic equation were used for calibration of the spectra, with assignment errors and average resolution of peaks highted in Table S2.†

### Data processing, analysis and visualisation

Peaks were selected using Bruker Data Analysis 4.4 after calibration, with parameters set to a signal to noise ratio of 4 and a minimum absolute intensity of 2 × 10^6^. The calibrated spectra included 6 decimal points and were exported in XML format and assigned using Formularity with a filter for elemental limits of C_0–100_H_0–200_O_0–30_N_0_S_0–2_P_0_ and an alignment tolerance of 0.5 ppm. Compounds were assigned by the lowest error against the Formularity Compound Identification Algorithm (CIA) database. The average assignment errors and resolutions of the assigned peaks from each spectrum can be found in Table S4.† Formularity output files were loaded into R 4.0.3 and processed to calculate heteroatomic classes, H/C & O/C ratios using tidyverse package collection. Ions detected in the negative control were subtracted from each experimental spectra to remove contaminating ions. Only ions which were present across all three replicates were utilised to calculate the average relative and absolute intensity values and standard deviations for each ion at each measured timepoint. Statistics were calculated using base functions in R 4.0.3. After calculating the change in relative intensity (RI), the results were passed through a filter to confirm that the ions changed by at least ±20% absolute intensity (AI), (if an ion changed in AI by ±20% within either treatment, abundance values were retained for both treatments to allow comparative analysis). Lignin-like compounds were defined as in previous studies, where ions which H/C = 0.7–1.5 and O/C = 0.1–0.7,^50,79,88–90^ were characterised as lignin-like. All graphs were created in R 4.0.3 using either ggplot or cowplot, with further annotation conducted in Adobe Illustrator CC. Further plots, raw, calibrated, assigned and accessory data, including Formularity filters and output, can be accessed *via* ESI† or the University of Edinburgh datashare at https://doi.org/10.7488/ds/3828.

### 2-D ^1^H–^13^C HSQC NMR

All samples were run on a He-cooled Bruker Avance III 600 MHz NMR spectrometer equipped with 5 mm TCI ^1^H/^13^C/^15^N CryoProbe™. Freeze dried samples were resuspended in 15 mL ddH_2_O prior to lignin precipitation using excess 2 M HCl, before collection under vacuum onto a glass fibre membrane. All samples were weighed prior to dissolution of 40 mg lignin in DMSO-d_6_ and sonication in a sonicating water bath, until full dissolution was achieved. One negative control sample only included less, approximately 20 mg of lignin, due to loss of mass during filtration. The prepared samples underwent a primary 1-D ^1^H NOESY presaturation experiment to determine the P1 pulse value (90° pulse width). The P1 value was utilised in to modify a standard ‘hsqcetgpsp.3’ experiment alongside a change in the offset frequency (O1P) value to 3.51 ppm to negate the PEG-like contaminant signal. Spectra were acquired over 4 scans with 50% NUS for a total experiment time of 58 minutes, with the spectra being processed using the qpol parameter. Peaks were identified using literature values^34,35,91–93^ and integrated using Bruker TopSpin 4.1 to retrieve a relative intensity level compared to the aromatic region.

## Data availability

All mass spectrometry and nuclear magnetic resonance datasets used in this study are available to download, in their original data formats, from the Horsfall Research Group repository at Edinburgh DataShare using https://doi.org/10.7488/ds/3828.

## Author contributions

Zak Towle: conceptualisation, methodology, software, validation, formal analysis, investigation, data curation, writing – original draft, writing – review & editing, visualisation. Faye Cruickshank: methodology, validation. Logan Mackay: methodology, validation, resources. David J. Clarke: conceptualisation, resources, writing – review & editing, supervision, project administration, funding acquisition. Louise E. Horsfall: conceptualisation, resources, writing – review & editing, supervision, project administration, funding acquisition.

## Conflicts of interest

There are no conflicts to declare.

## Supplementary Material

AN-149-D4AN00124A-s001

AN-149-D4AN00124A-s002

## References

[cit1] Zakzeski J., Bruijnincx P. C. A., Jongerius A. L., Weckhuysen B. M. (2010). Chem. Rev..

[cit2] Crestini C., Lange H., Sette M., Argyropoulos D. S. (2017). Green Chem..

[cit3] Vanholme R., Demedts B., Morreel K., Ralph J., Boerjan W. (2010). Plant Physiol..

[cit4] Fache M., Boutevin B., Caillol S. (2016). ACS Sustainable Chem. Eng..

[cit5] Schutyser W., Renders T., Van Den Bosch S., Koelewijn S. F., Beckham G. T., Sels B. F. (2018). Chem. Soc. Rev..

[cit6] Bugg T. D. H., Rahmanpour R. (2015). Curr. Opin. Chem. Biol..

[cit7] Salvachúa D., Karp E. M., Nimlos C. T., Vardon D. R., Beckham G. T. (2015). Green Chem..

[cit8] Rahmanpour R., Bugg T. D. H. (2015). Arch. Biochem. Biophys..

[cit9] Oates N. C., Abood A., Schirmacher A. M., Alessi A. M., Bird S. M., Bennett J. P., Leadbeater D. R., Li Y., Dowle A. A., Liu S., Tymokhin V. I., Ralph J., McQueen-Mason S. J., Bruce N. C. (2021). Proc. Natl. Acad. Sci. U. S. A..

[cit10] Liu H., Zhu L., Wallraf A. M., Räuber C., Grande P. M., Anders N., Gertler C., Werner B., Klankermayer J., Leitner W., Schwaneberg U. (2019). ACS Sustainable Chem. Eng..

[cit11] Kong W., Fu X., Wang L., Alhujaily A., Zhang J., Ma F., Zhang X., Yu H. (2017). Biotechnol. Biofuels.

[cit12] Zhang Z., Xia L., Wang F., Lv P., Zhu M., Li J., Chen K. (2015). Biotechnol. Biofuels.

[cit13] Kumar V. P., Sridhar M., Rao R. G. (2022). Sci. Rep..

[cit14] Zhu D., Liang N., Zhang R., Ahmad F., Zhang W., Yang B., Wu J., Geng A., Gabriel M., Sun J. (2020). ACS Sustainable Chem. Eng..

[cit15] Gutiérrez A., Rencoret J., Cadena E. M., Rico A., Barth D., del Río J. C., Martínez Á. T. (2012). Bioresour. Technol..

[cit16] Blánquez A., Ball A. S., González-Pérez J. A., Jiménez-Morillo N. T., González-Vila F., Arias M. E., Hernández M. (2017). PLoS One.

[cit17] Cao L., Lin L., Sui H., Wang H., Zhang Z., Jiao N., Zhou J. (2021). Green Chem..

[cit18] Baldrian P. (2006). FEMS Microbiol. Rev..

[cit19] Camarero S., García O., Vidal T., Colom J., del Río J. C., Gutiérrez A., Gras J. M., Monje R., Martínez M. J., Martínez Á. T. (2004). Enzyme Microb. Technol..

[cit20] Arias M. E., Arenas M., Rodríguez J., Soliveri J., Ball A. S., Hernández M. (2003). Appl. Environ. Microbiol..

[cit21] Khlifi R., Belbahri L., Woodward S., Ellouz M., Dhouib A., Sayadi S., Mechichi T. (2010). J. Hazard. Mater..

[cit22] Zeng S., Qin X., Xia L. (2017). Biochem. Eng. J..

[cit23] Wong Y., Yu J. (1999). Water Res..

[cit24] Wong D. W. S. (2009). Appl. Biochem. Biotechnol..

[cit25] Valli K., Wariishi H., Gold M. H. (1990). Biochemistry.

[cit26] Lahtinen M., Kruus K., Boer H., Kemell M., Andberg M., Viikari L., Sipilä J. (2009). J. Mol. Catal. B: Enzym..

[cit27] Yang J., Yang X., Lin Y., Ng T. B., Lin J., Ye X. (2015). PLoS One.

[cit28] Hilgers R., Van Dam A., Zuilhof H., Vincken J. P., Kabel M. A. (2020). ACS Catal..

[cit29] Hilgers R., Van Erven G., Boerkamp V., Sulaeva I., Potthast A., Kabel M. A., Vincken J. P. (2020). Green Chem..

[cit30] Hämäläinen V., Grönroos T., Suonpää A., Heikkilä M. W., Romein B., Ihalainen P., Malandra S., Birikh K. R. (2018). Front. Bioeng. Biotechnol..

[cit31] Wang L., Tan L., Hu L., Wang X., Koppolu R., Tirri T., van Bochove B., Ihalainen P., Seleenmary Sobhanadhas L. S., Seppälä J. V., Willför S., Toivakka M., Xu C. (2021). ACS Sustainable Chem. Eng..

[cit32] Lancefield C. S., Constant S., de Peinder P., Bruijnincx P. C. A. A., de Peinder P., Bruijnincx P. C. A. A., de Peinder P., Bruijnincx P. C. A. A. (2019). ChemSusChem.

[cit33] Boerjan W., Ralph J., Baucher M. (2003). Annu. Rev. Plant Biol..

[cit34] Lancefield C. S., Ojo O. S., Tran F., Westwood N. J. (2015). Angew. Chem..

[cit35] Tran F., Lancefield C. S., Kamer P. C. J., Lebl T., Westwood N. J. (2015). Green Chem..

[cit36] Montgomery J. R. D., Lancefield C. S., Miles-Barrett D. M., Ackermann K., Bode B. E., Westwood N. J., Lebl T. (2017). ACS Omega.

[cit37] Kishimoto T., Hiyama A., Yamashita A., Takano T., Tobimatsu Y., Urabe D. (2022). ACS Sustainable Chem. Eng..

[cit38] Chiang V. L., Funaoka M. (1990). Holzforschung.

[cit39] Jasiukaitytė-Grojzdek E., Huš M., Grilc M., Likozar B. (2020). Sci. Rep..

[cit40] Johansson A., Aaltonen O., Ylinen P. (1987). Biomass.

[cit41] Birikh K. , Michine A., Heikkilä M., Ihalainen P., Birikh K., Michine A., Heikkilä M. and Ihalainen P., in Biorefineries – Selected Processes, ed. K. Biernat, Intechopen, 2022, ch. 3, pp. 1 20

[cit42] Plavniece A., Dobele G., Djachkovs D., Jashina L., Bikovens O., Volperts A., Zhurinsh A. (2023). Materials.

[cit43] Brienza F., Cannella D., Montesdeoca D., Cybulska I., Debecker D. P. (2024). RSC Sustainability.

[cit44] Liao Y., de Beeck B. O., Thielemans K., Ennaert T., Snelders J., Dusselier M., Courtin C. M., Sels B. F. (2020). Mol. Catal..

[cit45] Sturgeon M. R., Kim S., Lawrence K., Paton R. S., Chmely S. C., Nimlos M., Foust T. D., Beckham G. T. (2014). ACS Sustainable Chem. Eng..

[cit46] Schutyser W., Renders T., Van Den Bosch S., Koelewijn S. F., Beckham G. T., Sels B. F. (2018). Chem. Soc. Rev..

[cit47] Qi Y., Fu P., Li S., Ma C., Liu C., Volmer D. A. (2020). Sci. Total Environ..

[cit48] Banoub J. H., Benjelloun-Mlayah B., Ziarelli F., Joly N., Delmas M. (2007). Rapid Commun. Mass Spectrom..

[cit49] Mikhael A., Fridgen T. D., Delmas M., Banoub J. (2021). J. Mass Spectrom..

[cit50] Echavarri-Bravo V., Tinzl M., Kew W., Cruickshank F., Logan Mackay C., Clarke D. J., Horsfall L. E. (2019). New Biotechnol..

[cit51] Andrianova A. A., DiProspero T., Geib C., Smoliakova I. P., Kozliak E. I., Kubátová A. (2018). J. Am. Soc. Mass Spectrom..

[cit52] Sander K., Dütsch L., Bremer M., Fischer S., Vogt C., Zuber J. (2023). Energy Fuels.

[cit53] Letourneau D. R., Volmer D. A. (2023). Mass Spectrom. Rev..

[cit54] Boes K. S., Roberts M. S., Vinueza N. R. (2018). J. Am. Soc. Mass Spectrom..

[cit55] Koch B. P., Ludwichowski K. U., Kattner G., Dittmar T., Witt M. (2008). Mar. Chem..

[cit56] Lv J., Zhang S., Wang S., Luo L., Cao D., Christie P. (2016). Environ. Sci. Technol..

[cit57] Leyva D., Jaffe R., Fernandez-Lima F. (2020). Anal. Chem..

[cit58] Corilo Y. E., Vaz B. G., Simas R. C., Lopes Nascimento H. D., Klitzke C. F., Pereira R. C. L., Bastos W. L., Santos Neto E. V., Rodgers R. P., Eberlin M. N. (2010). Anal. Chem..

[cit59] Fernandez-Lima F. A., Becker C., McKenna A. M., Rodgers R. P., Marshall A. G., Russell D. H. (2009). Anal. Chem..

[cit60] Klein G. C., Kim S., Rodgers R. P., Marshall A. G., Yen A. (2006). Energy Fuels.

[cit61] Cho E., Witt M., Hur M., Jung M. J., Kim S. (2017). Anal. Chem..

[cit62] Garcia J. S., Vaz B. G., Corilo Y. E., Ramires C. F., Saraiva S. A., Sanvido G. B., Schmidt E. M., Maia D. R. J., Cosso R. G., Zacca J. J., Eberlin M. N. (2013). Food Res. Int..

[cit63] Kew W., Goodall I., Clarke D., Uhrín D. (2017). J. Am. Soc. Mass Spectrom..

[cit64] Veličković D., Zhou M., Schilling J. S., Zhang J. (2021). J. Fungi.

[cit65] Terrell E., Garcia-Perez M. (2020). Energy Fuels.

[cit66] Paananen H., Eronen E., Mäkinen M., Jänis J., Suvanto M., Pakkanen T. T. (2020). Ind. Crops Prod..

[cit67] Owen B. C., Haupert L. J., Jarrell T. M., Marcum C. L., Parsell T. H., Abu-Omar M. M., Bozell J. J., Black S. K., Kenttämaa H. I. (2012). Anal. Chem..

[cit68] Terrell E., Carré V., Dufour A., Aubriet F. F., Le Brech Y., Garcia-Pérez M., Terrell E., Carre V., Doufour A., Aubriet F. F., Le Brech Y. (2020). ChemSusChem.

[cit69] Prothmann J., Spégel P., Sandahl M., Turner C. (2018). Anal. Bioanal. Chem..

[cit70] Qi Y., Hempelmann R., Volmer D. A. (2016). Anal. Bioanal. Chem..

[cit71] D'Auria M., Emanuele L., Racioppi R. (2012). Nat. Prod. Res..

[cit72] Moiseenko K. V., Glazunova O. A., Savinova O. S., Vasina D. V., Zherebker A. Y., Kulikova N. A., Nikolaev E. N., Fedorova T. V. (2021). Bioresour. Technol..

[cit73] McClelland D. J., Motagamwala A. H., Li Y., Rover M. R., Wittrig A. M., Wu C., Buchanan J. S., Brown R. C., Ralph J., Dumesic J. A., Huber G. W. (2017). Green Chem..

[cit74] Hilgers R., Vincken J. P., Gruppen H., Kabel M. A. (2018). ACS Sustainable Chem. Eng..

[cit75] Khatami S., Deng Y., Tien M., Hatcher P. G. (2019). J. Environ. Qual..

[cit76] Khatami S., Deng Y., Tien M., Hatcher P. G. (2019). Org. Geochem..

[cit77] Tolić N., Liu Y., Liyu A., Shen Y., Tfaily M. M., Kujawinski E. B., Longnecker K., Kuo L. J., Robinson E. W., Paša-Tolić L., Hess N. J. (2017). Anal. Chem..

[cit78] Qi Y., Hempelmann R., Volmer D. A. (2016). Anal. Bioanal. Chem..

[cit79] Kim S., Kramer R. W., Hatcher P. G. (2003). Anal. Chem..

[cit80] Kamaya Y., Nakatsubo F., Higuchi T., Iwahara S. (1981). Arch. Microbiol..

[cit81] Kamaya Y., Nakatsubo F., Higuchi T. (1983). Agric. Biol. Chem..

[cit82] Guo H., Miles-Barrett D. M., Neal A. R., Zhang T., Li C., Westwood N. J. (2018). Chem. Sci..

[cit83] Mnich E., Vanholme R., Oyarce P., Liu S., Lu F., Goeminne G., Jørgensen B., Motawie M. S., Boerjan W., Ralph J., Ulvskov P., Møller B. L., Bjarnholt N., Harholt J. (2017). Plant Biotechnol. J..

[cit84] Constant S., Wienk H. L. J., Frissen A. E., De Peinder P., Boelens R., Van Es D. S., Grisel R. J. H., Weckhuysen B. M., Huijgen W. J. J., Gosselink R. J. A., Bruijnincx P. C. A. (2016). Green Chem..

[cit85] Morreel K., Kim H., Lu F., Dima O., Akiyama T., Vanholme R., Niculaes C., Goeminne G., Inzé D., Messens E., Ralph J., Boerjan W. (2010). Anal. Chem..

[cit86] Sim H.-J., Kim J., Lee K., Hong J. (2013). Molecules.

[cit87] Sanz M., De Simón B. F., Cadahía E., Esteruelas E., Muñoz A. M., Hernández T., Estrella I., Pinto E. (2012). J. Mass Spectrom..

[cit88] Hockaday W. C., Purcell J. M., Marshall A. G., Baldock J. A., Hatcher P. G. (2009). Limnol. Oceanogr.: Methods.

[cit89] Liu S., He Z., Tang Z., Liu L., Hou J., Li T., Zhang Y., Shi Q., Giesy J. P., Wu F. (2020). Sci. Total Environ..

[cit90] Rivas-Ubach A., Liu Y., Bianchi T. S., Tolić N., Jansson C., Paša-Tolić L. (2018). Anal. Chem..

[cit91] Ralph S. and Ralph J.

[cit92] Del Río J. C., Rencoret J., Prinsen P., Martínez Á. T., Ralph J., Gutiérrez A. (2012). J. Agric. Food Chem..

[cit93] Van Den Bosch S., Renders T., Kennis S., Koelewijn S. F., Van Den Bossche G., Vangeel T., Deneyer A., Depuydt D., Courtin C. M., Thevelein J. M., Schutyser W., Sels B. F. (2017). Green Chem..

