# Supplementary material for: Utilising Fourier transform ion cyclotron resonance mass spectrometry (FT-ICR MS) to track the oxidation of lignin by an alkaliphilic laccase
Source: Analyst. 2024 Mar 8;149(8):2399–411. doi: 10.1039/d4an00124a (PMC11018093; doi:10.1039/d4an00124a)
Supplement: AN-149-D4AN00124A-s002 [file AN-149-D4AN00124A-s002.pdf]

Electronic Supplementary Material (ESI) for Analyst.
This journal is © The Royal Society of Chemistry 2024

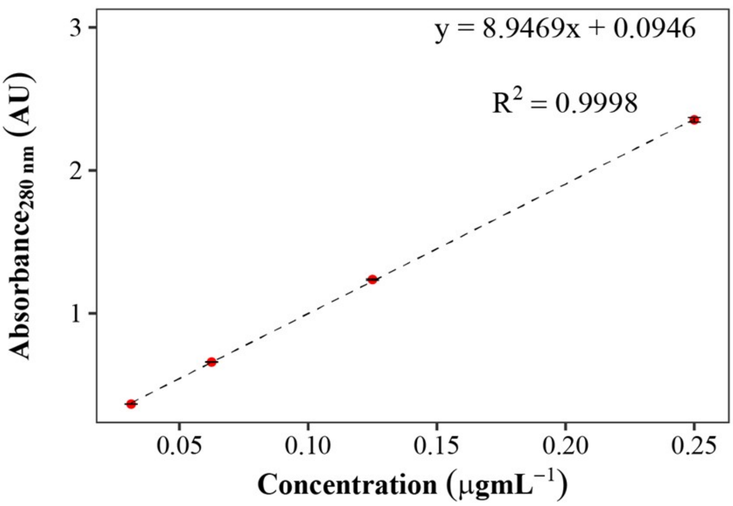

Figure S1, Standard curve of different dilutions of Sigma Kraft Lignin (SKL). Each sample was tested in triplicate to ensure that the calibration curve was sufficient, error bars of the sample represent the standard error of each lignin concentration tested.

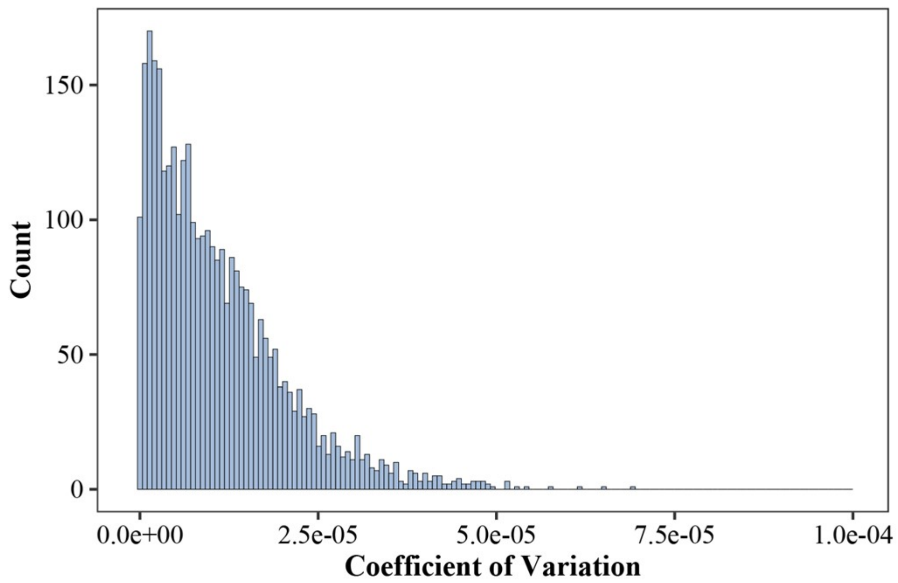

Figure S2, The calculated coefficient of variation (CoV) for the quality control samples. The variation is left-skewed and extremely low (between 0 and 7.5×10^{-5}) indicating that there is minimal variation between the same peaks of the three QC replicates.

Table S1, Number of ions detected by the FT-ICR MS experiment, and the number of ions assigned by Formularity.

Time	Replicate	Treatment	Total Number of Ions	Number of Ions Assigned	Assigned Ions (%)
0	1	Enzyme-Only Control	828	588	71.0
2	1	Enzyme-Only Control	903	648	71.8
24	1	Enzyme-Only Control	823	597	72.5
0	1	Negative Control	9265	5691	61.4
0	2	Negative Control	9756	5752	59.0
0	3	Negative Control	9356	5478	58.6
2	1	Negative Control	8778	5300	60.4
2	2	Negative Control	9609	5633	58.6
2	3	Negative Control	9489	5485	57.8
24	1	Negative Control	9639	5621	58.3
24	2	Negative Control	9417	5487	58.3
24	3	Negative Control	9262	5425	58.6
0	1	Laccase treated	9877	5894	59.7
0	2	Laccase treated	9790	5778	59.0
0	3	Laccase treated	9799	5713	58.3
2	1	Laccase treated	9022	5150	57.1
2	2	Laccase treated	9375	5317	56.7
2	3	Laccase treated	9243	5225	56.5
24	1	Laccase treated	7920	4193	52.9
24	2	Laccase treated	8122	4256	52.4
24	3	Laccase treated	8237	4298	52.2

Table S2, Count data for each mass bracket shown in Figure 3, counts and standard deviations are rounded to the nearest whole number.

Mass Bracket (Da)	Treatment	Time (Hrs)	Mean Count	Standard Deviation
140-250	Negative Control	0	75	5
140-250	Negative Control	2	77	5
140-250	Negative Control	24	79	1
140-250	Laccase-treated	0	84	2
140-250	Laccase-treated	2	80	3
140-250	Laccase-treated	24	77	2
250-350	Negative Control	0	428	17
250-350	Negative Control	2	428	14
250-350	Negative Control	24	432	5
250-350	Laccase-treated	0	447	8
250-350	Laccase-treated	2	438	4
250-350	Laccase-treated	24	424	5
350-450	Negative Control	0	703	16
350-450	Negative Control	2	702	15
350-450	Negative Control	24	710	8
350-450	Laccase-treated	0	739	17
350-450	Laccase-treated	2	706	13
350-450	Laccase-treated	24	673	16
450-550	Negative Control	0	824	19
450-550	Negative Control	2	825	18
450-550	Negative Control	24	828	15
450-550	Laccase-treated	0	874	26
450-550	Laccase-treated	2	835	13
450-550	Laccase-treated	24	755	4
550-650	Negative Control	0	637	37
550-650	Negative Control	2	619	19
550-650	Negative Control	24	618	3
550-650	Laccase-treated	0	646	23
550-650	Laccase-treated	2	582	15
550-650	Laccase-treated	24	485	9
650-750	Negative Control	0	460	17
650-750	Negative Control	2	456	10
650-750	Negative Control	24	452	17
650-750	Laccase-treated	0	443	10
650-750	Laccase-treated	2	417	8
650-750	Laccase-treated	24	305	6
750-850	Negative Control	0	332	4
750-850	Negative Control	2	327	11
750-850	Negative Control	24	327	5
750-850	Laccase-treated	0	322	5
750-850	Laccase-treated	2	286	5
750-850	Laccase-treated	24	204	6

850-950	Negative Control	0	270	8
850-950	Negative Control	2	246	17
850-950	Negative Control	24	255	12
850-950	Laccase-treated	0	246	3
850-950	Laccase-treated	2	198	4
850-950	Laccase-treated	24	112	6
950-1050	Negative Control	0	182	12
950-1050	Negative Control	2	157	19
950-1050	Negative Control	24	161	7
950-1050	Laccase-treated	0	173	15
950-1050	Laccase-treated	2	130	6
950-1050	Laccase-treated	24	33	3
1050-1150	Negative Control	0	69	18
1050-1150	Negative Control	2	49	10
1050-1150	Negative Control	24	56	4
1050-1150	Laccase-treated	0	64	2
1050-1150	Laccase-treated	2	35	4
1050-1150	Laccase-treated	24	8	2
1150-1250	Negative Control	0	22	7
1150-1250	Negative Control	2	10	6
1150-1250	Negative Control	24	12	3
1150-1250	Laccase-treated	0	15	4
1150-1250	Laccase-treated	2	6	2
1150-1250	Laccase-treated	24	2	1
>1250	Negative Control	0	4	3
>1250	Negative Control	2	3	3
>1250	Negative Control	24	5	1
>1250	Laccase-treated	0	3	2
>1250	Laccase-treated	2	4	1
>1250	Laccase-treated	24	3	2

Table S3, Compounds used for spectra calibration of the digest samples and controls, the asterisk (*) signifies compounds belonging to the identified polymer group.

Number	Molecular Formulae	*m/z* [M-H]$^-$ (Da)	Number	Molecular Formulae	*m/z* [M-H]$^-$ (Da)
1	$C_{10}H_9O_6$	225.0405	33	$C_{20}H_{19}O_7$	371.1136
2	$C_{16}H_{29}O_2$	253.2173	34	$C_{20}H_{21}O_7$	373.1293
3	$C_{16}H_{31}O_2$	255.2330	35	$C_{18}H_{19}O_9$	379.1035
4	$C_{16}H_{15}O_4$	271.0976	36	$C_{20}H_{21}O_8$	389.1242
5	$C_{18}H_{31}O_2$	279.2330	37	$C_{21}H_{41}O_6$	389.2909
6	$C_{18}H_{33}O_2$	281.2486	38	$C_{22}H_{37}O_6^*$	397.2596
7	$C_{18}H_{35}O_2$	283.2643	39	$C_{21}H_{21}O_8$	401.1242
8	$C_{16}H_{29}O_4$	285.2071	40	$C_{22}H_{23}O_8$	415.1398
9	$C_{14}H_{29}O_4S$	293.1792	41	$C_{22}H_{25}O_8$	417.1555
10	$C_{14}H_{17}O_7$	297.0980	42	$C_{21}H_{23}O_9$	419.1348
11	$C_{16}H_{25}O_3S$	297.1530	43	$C_{22}H_{25}O_9$	433.1504
12	$C_{20}H_{27}O_2$	299.2017	44	$C_{21}H_{23}O_{10}$	435.1297
13	$C_{16}H_{13}O_6$	301.0718	45	$C_{20}H_{23}O_{11}$	439.1246
14	$C_{16}H_{15}O_6$	303.0874	46	$C_{24}H_{41}O_7^*$	441.2858
15	$C_{17}H_{27}O_3S$	311.1686	47	$C_{22}H_{25}O_{10}$	449.1453
16	$C_{18}H_{31}O_4$	311.2228	48	$C_{26}H_{45}O_8^*$	485.3120
17	$C_{19}H_{21}O_4$	313.1445	49	$C_{28}H_{49}O_9^*$	529.3382
18	$C_{12}H_{17}O_{10}$	321.0827	50	$C_{30}H_{53}O_{10}^*$	573.3644
19	$C_{18}H_{29}O_3S$	325.1843	51	$C_{31}H_{37}O_{11}$	585.2341
20	$C_{18}H_{31}O_5$	327.2177	52	$C_{32}H_{57}O_{11}^*$	617.3906
21	$C_{18}H_{33}O_5$	329.2333	53	$C_{33}H_{59}O_{12}$	647.4012
22	$C_{17}H_{15}O_7$	331.0823	54	$C_{34}H_{61}O_{12}$	661.4169
23	$C_{12}H_{19}O_{11}$	339.0933	55	$C_{34}H_{61}O_{13}^*$	677.4118
24	$C_{19}H_{31}O_3S$	339.1999	56	$C_{36}H_{65}O_{13}^*$	705.4431
25	$C_{19}H_{17}O_6$	341.1031	57	$C_{35}H_{65}O_{14}$	709.4380
26	$C_{12}H_{21}O_{11}$	341.1089	58	$C_{38}H_{69}O_{14}^*$	749.4693
27	$C_{18}H_{33}O_6$	345.2283	59	$C_{40}H_{73}O_{15}^*$	793.4955
28	$C_{20}H_{33}O_5$	353.2333	60	$C_{42}H_{77}O_{16}^*$	837.5217
29	$C_{19}H_{19}O_7$	359.1136	61	$C_{44}H_{81}O_{17}^*$	881.5479
30	$C_{18}H_{17}O_8$	361.0929	62	$C_{46}H_{85}O_{18}^*$	925.5741
31	$C_{18}H_{19}O_8$	363.1085	63	$C_{48}H_{89}O_{19}^*$	969.6004
32	$C_{24}H_{47}O_2$	367.3582			

Formularity Assignment:

The parameters used for assignment in Formularity can be found in the electronic supplementary materials at https://doi.org/10.7488/ds/3828. Briefly, Charge was set to 1, Ionization as proton_detachment [M-p(negative)], with an alignment and formula tolerance of 0.500 ppm and a DB mass limit of 1000. Formulae were assigned using the lowest score achieved with Formula building blocks of CH2, H2 and O included with a maximum relationship gap of 5 and atomic mass unit (AMU) error of 0.00002. A user defined filter was set as O <30 AND C <=100 AND N =0 AND P=0 AND S<=2.

Table S4, Average Resolution and Assignment Error of Spectra Assigned using *Formularity*.

Treatment (Replicate)	Time (Hrs)	Average Resolution	Assignment Error (±ppb)
Negative Control (1)	0	376,167	182
Negative Control (2)	0	387,110	184
Negative Control (3)	0	383,862	182
Negative Control (1)	2	381,063	184
Negative Control (2)	2	384,027	184
Negative Control (3)	2	384,314	183
Negative Control (1)	24	380,011	183
Negative Control (2)	24	385,673	184
Negative Control (3)	24	385,744	185
Laccase (1)	0	385,873	184
Laccase (2)	0	384,377	183
Laccase (3)	0	384,526	184
Laccase (1)	2	393,855	187
Laccase (2)	2	392,296	187
Laccase (3)	2	392,678	186
Laccase (1)	24	411,514	194
Laccase (2)	24	411,155	196
Laccase (3)	24	410,366	194

Table S5 ANOVA and Post-hoc Tukey Analysis Tables for Ion Count Stats

ANOVA

> aov2 <- aov(n ~ Time * Treatment, data = TP2Tx)

	Df	Sum Sq	Mean Sq	F value	Pr(<F)	
**Time**	2	829159	414579	62.33	4.58E-07	***
**Treatment**	1	482489	482489	72.54	1.97E-06	***
**Time:Treatment**	2	660961	330480	49.69	1.56E-06	***
**Residuals**	12	79818	6652			

Signif. codes: 0 ‘***’ 0.001 ‘**’ 0.01 ‘*’ 0.05 ‘.’ 0.1 ‘ ’ 1

TukeyHSD

Tukey multiple comparisons of means

95% family-wise confidence level

Fit: aov(formula = n ~ Time * Treatment, data = TP2Tx)

Signif. codes: 0 ‘***’ 0.001 ‘**’ 0.01 ‘*’ 0.05 ‘.’ 0.1 ‘ ’ 1

Time	diff	lwr	upr	p adj	significance
T2-T0	-223.333	-348.955	-97.7122	0.0012821	**
T24-T0	-523.833	-649.455	-398.212	0.0000003	***
T24-T2	-300.5	-426.121	-174.879	0.000096	***
**Treatment**	diff	lwr	upr	p adj	
LaccaseTreated-NegativeControl	-327.444	-411.212	-243.677	2.00E-06	***
**Time:Treatment**	diff	lwr	upr	p adj	
2:Negative-0:Negative	-106.333	-330.007	117.3399	0.6152693	
24:Negative-0:Negative	-71.6667	-295.34	152.0065	0.881699	
24:Negative-2:Negative	34.66667	-189.007	258.3399	0.9942304	
2:Positive-0:Positive	-340.333	-564.007	-116.66	0.0027071	**
24:Positive-0:Positive	-976	-1199.67	-752.327	0.0000001	***
24:Positive-2:Positive	-635.667	-859.34	-411.993	0.0000069	***
0:Positive-0:Negative	52	-171.673	275.6732	0.9656999	
2:Positive-0:Negative	-288.333	-512.007	-64.6601	0.0097396	**
24:Positive-0:Negative	-924	-1147.67	-700.327	0.0000001	***
0:Positive-2:Negative	158.3333	-65.3399	382.0065	0.2375494	
2:Positive-2:Negative	-182	-405.673	41.6732	0.138829	
24:Positive-2:Negative	-817.667	-1041.34	-593.993	0.0000004	***
0:Positive-24:Negative	123.6667	-100.007	347.3399	0.4690461	
2:Positive-24:Negative	-216.667	-440.34	7.006528	0.0596045	.
24:Positive-24:Negative	-852.333	-1076.01	-628.66	0.0000003	***

Table S6 Poisson GLM results between Time 0 and Time 2 and 24. ns represents no significant difference between Treatment, Time points in each mass bracket, with $p < 0.1$ '.', < 0.05 '*', < 0.01 '**',< 0.001 '***'.

T0:T2 - T0:T24<br>Laccase vs Negative Control<br>Poisson Model	Estimate	Std. Error	z value	Pr(>|z|)	
Laccase – Time 2 – 140-250 m/z	-0.06697	0.130026	-0.51507	0.606501	ns
Laccase – Time 24 – 140-250 m/z	-0.13933	0.130368	-1.06877	0.285172	ns
Laccase – Time 2 – 250-350 m/z	-0.01953	0.055352	-0.35279	0.724247	ns
Laccase – Time 24 – 250-350 m/z	-0.06132	0.055513	-1.10465	0.269313	ns
Laccase – Time 2 – 350-450 m/z	-0.04473	0.043271	-1.03374	0.301258	ns
Laccase – Time 24 – 350-450 m/z	-0.10346	0.043474	-2.37982	0.017321	*
Laccase – Time 2 – 450-550 m/z	-0.04725	0.039863	-1.18528	0.235906	ns
Laccase – Time 24 – 450-550 m/z	-0.15203	0.040378	-3.76518	0.000166	***
Laccase – Time 2 – 550-650 m/z	-0.07562	0.046361	-1.63117	0.102854	ns
Laccase – Time 24 – 550-650 m/z	-0.25636	0.047596	-5.38618	7.20E-08	***
Laccase – Time 2 – 650-750 m/z	-0.05183	0.054841	-0.94501	0.344656	ns
Laccase – Time 24 – 650-750 m/z	-0.35463	0.057491	-6.16855	6.89E-10	***
Laccase – Time 2 – 750-850 m/z	-0.10454	0.065019	-1.60777	0.107886	ns
Laccase – Time 24 – 750-850 m/z	-0.44124	0.068517	-6.43986	1.20E-10	***
Laccase – Time 2 – 850-950 m/z	-0.12353	0.075012	-1.64674	0.099611	.
Laccase – Time 24 – 850-950 m/z	-0.73123	0.082987	-8.81128	1.24E-18	***
Laccase – Time 2 – 950-1050 m/z	-0.14432	0.091934	-1.56985	0.11645	ns
Laccase – Time 24 – 950-1050 m/z	-1.52789	0.125811	-12.1444	6.15E-34	***
Laccase – Time 2 – 1050-1150 m/z	-0.26803	0.162234	-1.65211	0.098512	.
Laccase – Time 24 – 1050-1150 m/z	-1.82389	0.236701	-7.70548	1.30E-14	***
Laccase – Time 2 – 1150-1250 m/z	-0.13118	0.356598	-0.36786	0.712977	ns
Laccase – Time 24 – 1150-1250 m/z	-1.29186	0.455806	-2.83424	0.004593	**
Laccase – Time 2 – >1250 m/z	0.519124	0.646474	0.803008	0.42197	ns
Laccase – Time 24 – >1250 m/z	-0.35895	0.631228	-0.56865	0.569597	ns

Table S7 Poisson GLM results between Time 2 and Time 0 and 24. ns represents no significant difference between Treatment, Time points in each mass bracket, with p < 0.1 '.', < 0.05 '*', < 0.01 '**',< 0.001 '***'.

T2:T0 - T2:T24<br>Laccase vs Negative Control<br>Poisson Model	Estimate	Std. Error	z value	Pr(>|z|)	
Laccase – Time 0 – 140-250 m/z	0.066973	0.130026	0.515074	0.606501	ns
Laccase – Time 24 – 140-250 m/z	-0.07236	0.130559	-0.55423	0.579419	ns
Laccase – Time 0 – 250-350 m/z	0.019527	0.055352	0.352788	0.724247	ns
Laccase – Time 24 – 250-350 m/z	-0.0418	0.055667	-0.7508	0.452771	ns
Laccase – Time 0 – 350-450 m/z	0.044731	0.043271	1.03374	0.301258	ns
Laccase – Time 24 – 350-450 m/z	-0.05873	0.043726	-1.34311	0.179235	ns
Laccase – Time 0 – 450-550 m/z	0.047248	0.039863	1.185282	0.235906	ns
Laccase – Time 24 – 450-550 m/z	-0.10478	0.04059	-2.58149	0.009837	**
Laccase – Time 0 – 550-650 m/z	0.075623	0.046361	1.631172	0.102854	ns
Laccase – Time 24 – 550-650 m/z	-0.18074	0.048345	-3.73849	0.000185	***
Laccase – Time 0 – 650-750 m/z	0.051825	0.054841	0.945005	0.344656	ns
Laccase – Time 24 – 650-750 m/z	-0.30281	0.057962	-5.22425	1.75E-07	***
Laccase – Time 0 – 750-850 m/z	0.104536	0.065019	1.607768	0.107886	ns
Laccase – Time 24 – 750-850 m/z	-0.33671	0.069582	-4.83897	1.31E-06	***
Laccase – Time 0 – 850-950 m/z	0.123525	0.075012	1.646743	0.099611	.
Laccase – Time 24 – 850-950 m/z	-0.6077	0.085603	-7.09908	1.26E-12	***
Laccase – Time 0 – 950-1050 m/z	0.144322	0.091934	1.569849	0.11645	ns
Laccase – Time 24 – 950-1050 m/z	-1.38357	0.129477	-10.6858	1.19E-26	***
Laccase – Time 0 – 1050-1150 m/z	0.268029	0.162234	1.652112	0.098512	.
Laccase – Time 24 – 1050-1150 m/z	-1.55587	0.249537	-6.23502	4.52E-10	***
Laccase – Time 0 – 1150-1250 m/z	0.131178	0.356598	0.36786	0.712977	ns
Laccase – Time 24 – 1150-1250 m/z	-1.16068	0.510562	-2.27335	0.023005	.
Laccase – Time 0 – >1250 m/z	-0.51912	0.646474	-0.80301	0.42197	ns
Laccase – Time 24 – >1250 m/z	-0.87807	0.642135	-1.36742	0.171493	ns

Table S8. Poisson GLM results between Time 0 and Time 2 and 24 for heteroatomic class of CHO_x compounds. ns represents no significant difference between Treatment, Time points in each oxygen number, with p < 0.1 ‘.’, < 0.05 ‘*’, < 0.01 ‘**’,< 0.001 ‘***’.

	Estimate	Std. Error	z value	Pr(>|z|)	Significance
TreatmentPositive:Time24:OClassO2	-0.21869	0.576663	-0.37923	0.704515	
TreatmentPositive:Time24:OClassO3	-0.37648	0.299743	-1.256	0.209116	
TreatmentPositive:Time24:OClassO4	-0.26437	0.18276	-1.44654	0.148027	
TreatmentPositive:Time24:OClassO5	-0.25895	0.133641	-1.93763	0.052669	.
TreatmentPositive:Time24:OClassO6	-0.27753	0.11619	-2.3886	0.016912	*
TreatmentPositive:Time24:OClassO7	-0.25994	0.103785	-2.50465	0.012257	*
TreatmentPositive:Time24:OClassO8	-0.25671	0.102409	-2.50676	0.012184	*
TreatmentPositive:Time24:OClassO9	-0.33473	0.100371	-3.33491	0.000853	***
TreatmentPositive:Time24:OClassO10	-0.3099	0.096822	-3.20073	0.001371	**
TreatmentPositive:Time24:OClassO11	-0.43014	0.095333	-4.51198	6.42E-06	***
TreatmentPositive:Time24:OClassO12	-0.40866	0.096051	-4.25462	2.09E-05	***
TreatmentPositive:Time24:OClassO13	-0.36989	0.094299	-3.92251	8.76E-05	***
TreatmentPositive:Time24:OClassO14	-0.32638	0.098759	-3.30475	0.000951	***
TreatmentPositive:Time24:OClassO15	-0.28214	0.104167	-2.70854	0.006758	**
TreatmentPositive:Time24:OClassO16	-0.39235	0.115004	-3.41166	0.000646	***
TreatmentPositive:Time24:OClassO17	-0.53684	0.127114	-4.22332	2.41E-05	***
TreatmentPositive:Time24:OClassO18	-0.66733	0.153965	-4.33428	1.46E-05	***
TreatmentPositive:Time24:OClassO19	-0.79273	0.18755	-4.22679	2.37E-05	***
TreatmentPositive:Time24:OClassO20	-0.68418	0.209284	-3.26914	0.001079	**
TreatmentPositive:Time24:OClassO21	-0.7263	0.248152	-2.92684	0.003424	**
TreatmentPositive:Time24:OClassO22	-2.16613	0.556928	-3.88944	0.0001	***
TreatmentPositive:Time24:OClassO23	-1.37948	0.44655	-3.08919	0.002007	**
TreatmentPositive:Time24:OClassO24	0.269664	0.686759	0.392661	0.69457	
TreatmentPositive:Time24:OClassO25	-1.38629	1.554563	-0.89176	0.372523	

Table S9. Poisson GLM results between Time 0 and Time 2 and 24 for heteroatomic class of CHO_xS_2 compounds. ns represents no significant difference between Treatment, Time points in each oxygen number, with p < 0.1 '.', < 0.05 '*', < 0.01 '**',< 0.001 '***'.

	Estimate	Std. Error	z value	Pr(>|z|)	
TreatmentPositive:Time24:OClassO3	0.05001	0.332622	0.150352	0.880487	
TreatmentPositive:Time24:OClassO4	0.227784	0.298987	0.761852	0.446149	
TreatmentPositive:Time24:OClassO5	0.251314	0.310239	0.810067	0.417901	
TreatmentPositive:Time24:OClassO6	-0.08004	0.389303	-0.20561	0.837099	
TreatmentPositive:Time24:OClassO7	-0.30228	0.455593	-0.66349	0.507017	
TreatmentPositive:Time24:OClassO8	-0.59784	0.831209	-0.71924	0.471994	
TreatmentPositive:Time24:OClassO9	-0.94504	0.649015	-1.45611	0.145361	
TreatmentPositive:Time24:OClassO10	-0.96854	0.314784	-3.07684	0.002092	**
TreatmentPositive:Time24:OClassO11	-0.76447	0.234247	-3.26353	0.0011	**
TreatmentPositive:Time24:OClassO12	-0.74712	0.199496	-3.74503	0.00018	***
TreatmentPositive:Time24:OClassO13	-0.4639	0.172398	-2.69089	0.007126	**
TreatmentPositive:Time24:OClassO14	-0.50493	0.168401	-2.99836	0.002714	**
TreatmentPositive:Time24:OClassO15	-0.55962	0.194828	-2.87236	0.004074	**
TreatmentPositive:Time24:OClassO16	-0.50441	0.216218	-2.33288	0.019655	*
TreatmentPositive:Time24:OClassO17	-0.70779	0.298743	-2.36923	0.017825	*
TreatmentPositive:Time24:OClassO18	-0.34645	0.395834	-0.87523	0.381446	
TreatmentPositive:Time24:OClassO19	-0.09353	0.430286	-0.21736	0.82793	
TreatmentPositive:Time24:OClassO20	-1.09861	1.027402	-1.06931	0.28493	

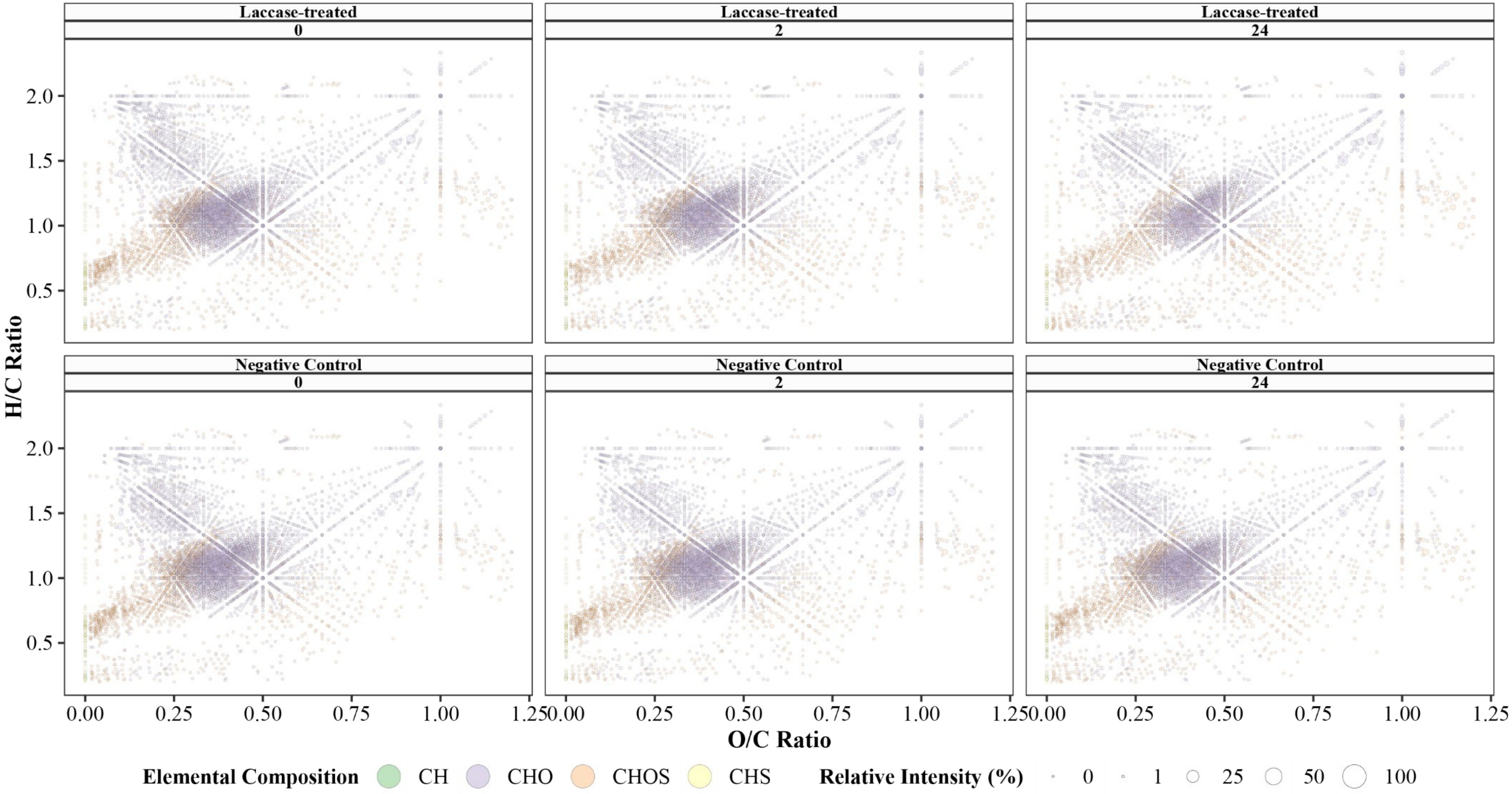

Figure S3. van Krevelen Diagram displaying the relative intensity of each ion in both treatments across the experimental time course, where the size of each point represents the intensity of the ion. Ions are coloured according to their elemental composition. Each van Krevelen diagram is similar, although there is a lower density of ions in the lignin-like region (H/C ratio = 1.15 & O/C ratio = 0.375) after 24 hours of laccase treatment, with no loss in ions observed in the negative control.

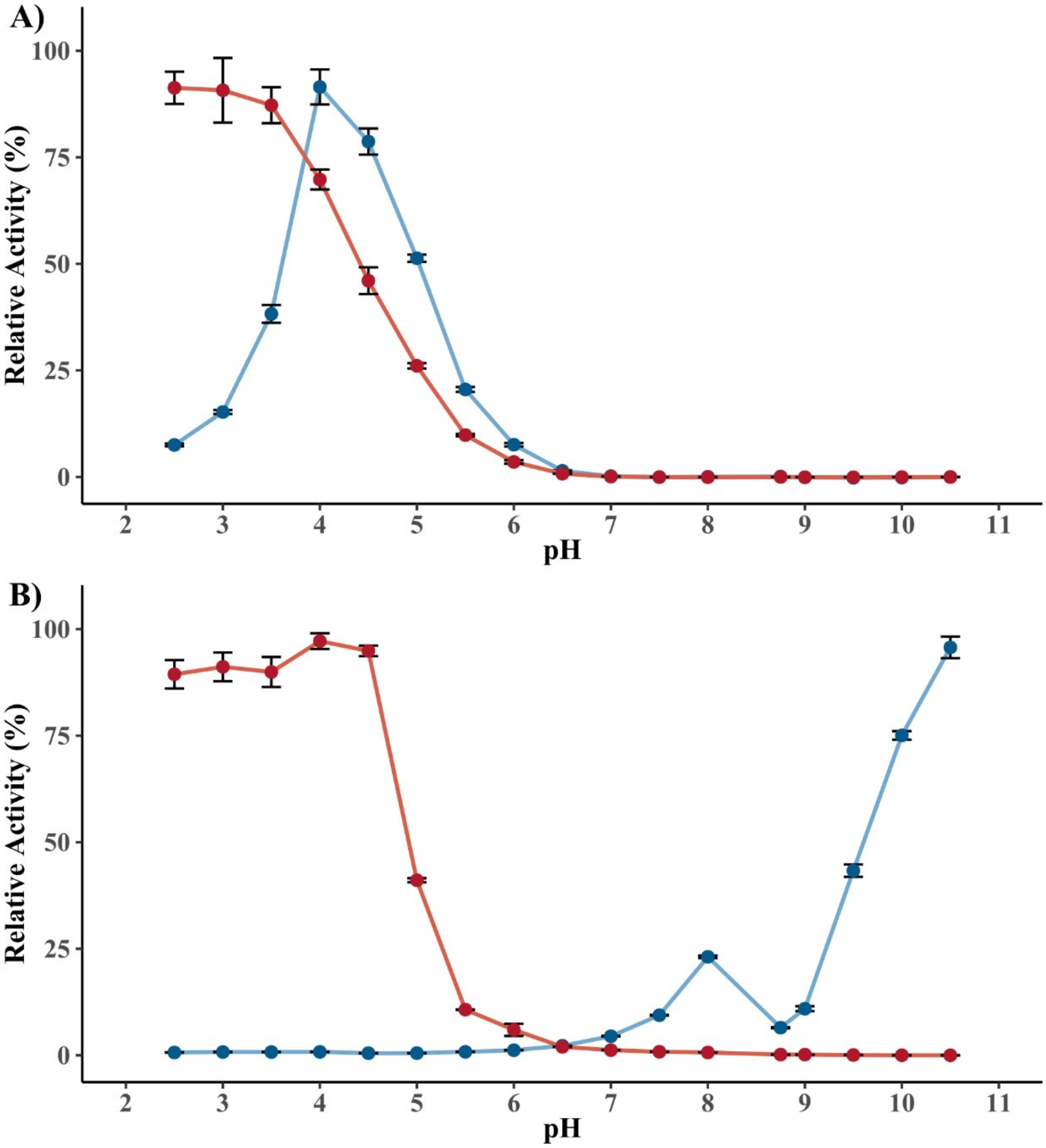

Figure S4, Activity of an acidic-active laccase from *Panus rudis (PrL)*, expressed in *Pichia pastoris* (A) and MetZyme® laccase (B) across a range of pH values from 2.5 to 10.5From pH 2.5–8.0 a McIlvaine buffer (20 mM Citrate:Phosphate) was used, above pH 8.0 Glycine:HCl was used. MetZyme® displayed an optimum pH against 2,6-DMP above pH 10.5 as expected, however, the optimum pH for ABTS was < pH 4.5, further indicating the increased ability of ABTS to oxidise at lower pHs.

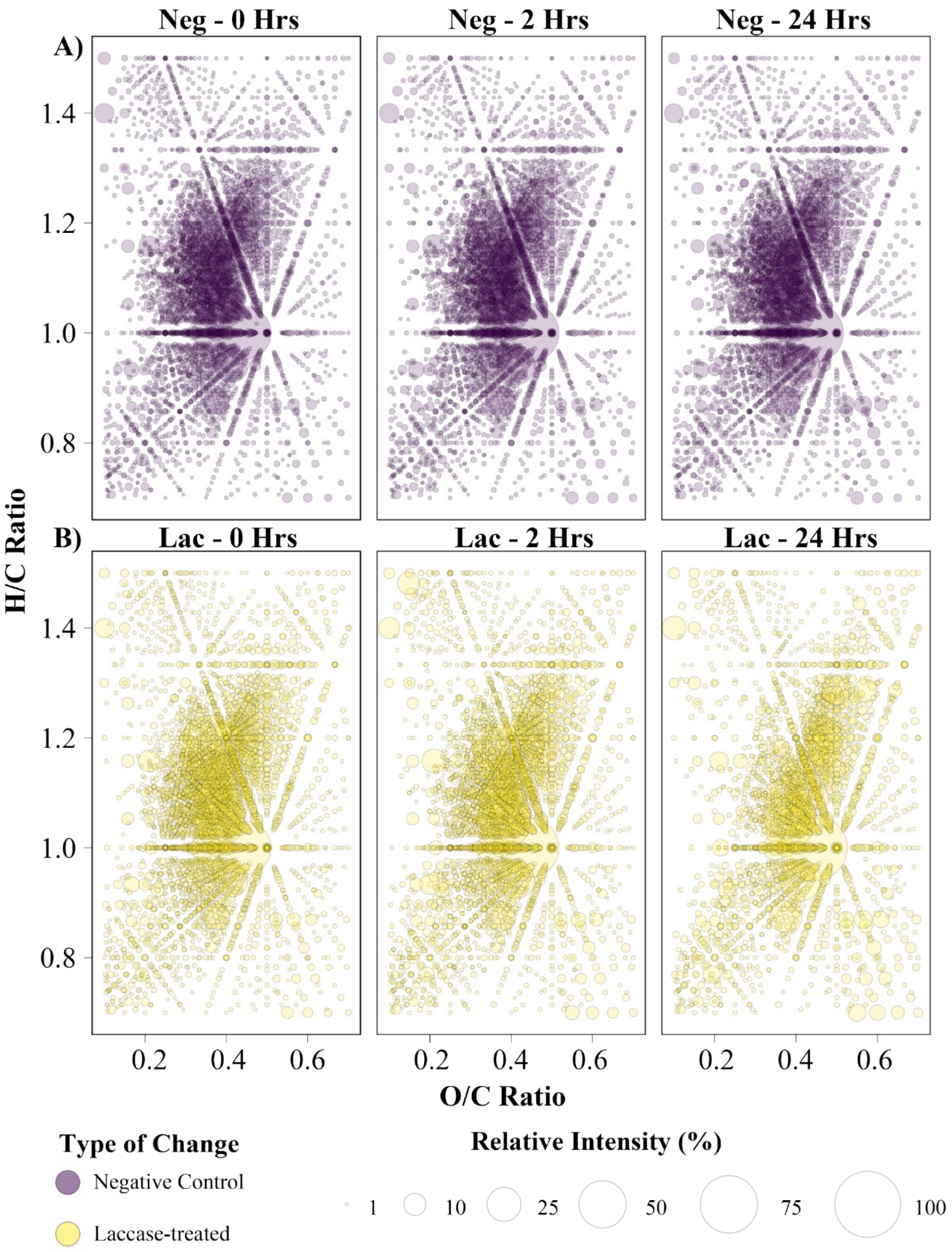

Figure S5. van Krevelen Diagram of RI over the time course. A) The negative control treatment. B) The laccase-treated sample.

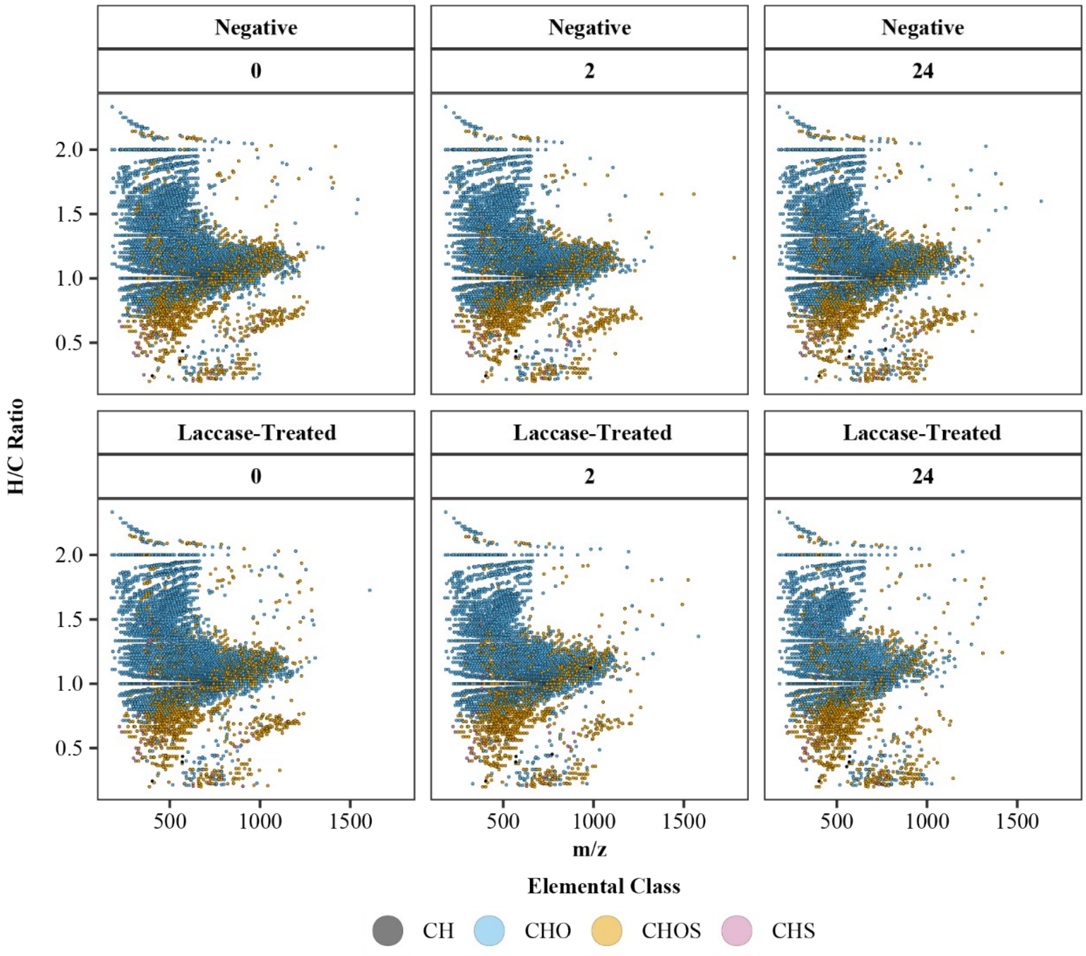

Figure S6. H/C ratio vs m/z plot for the negative control and laccase-treated samples over time.

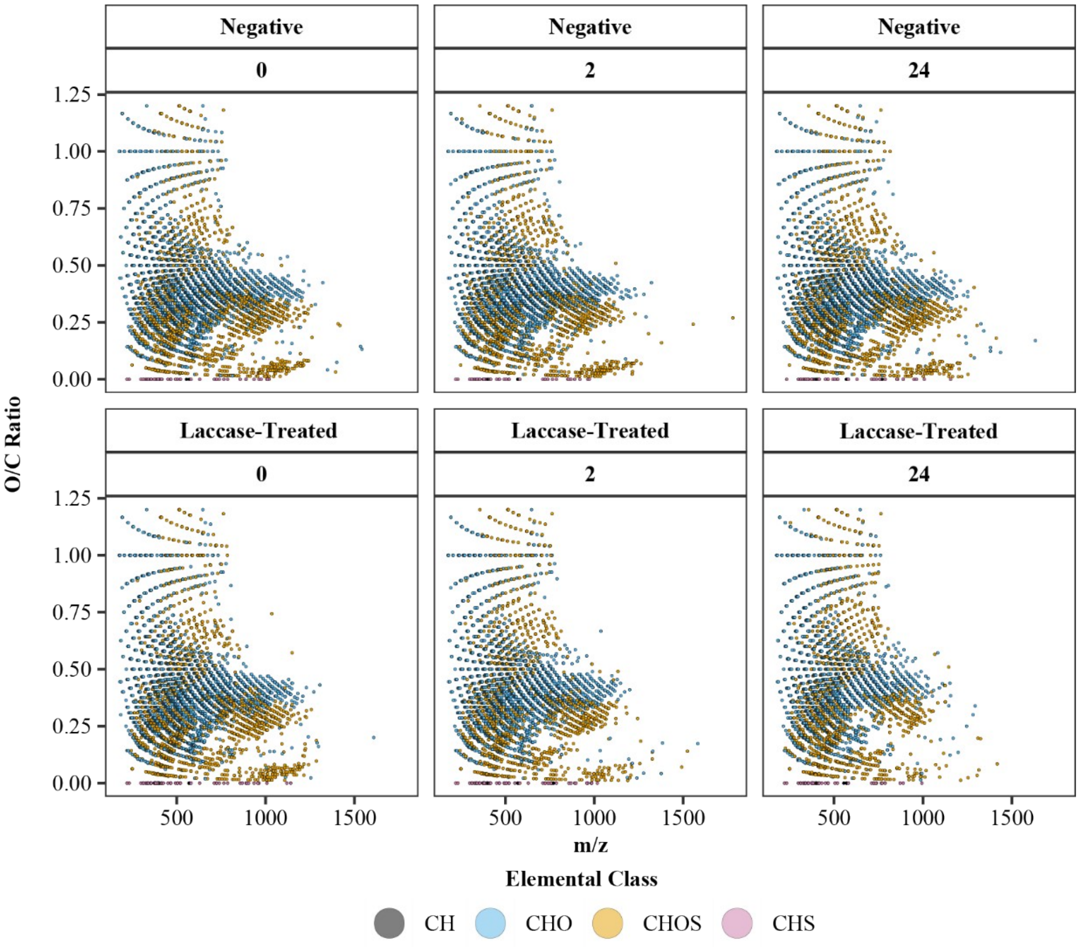

Figure S7. H/C ratio vs m/z plot for the negative control and laccase-treated samples over time.

**Laccase 1**					**Laccase 2**				**Laccase 3**			
**Object**	**Integral [abs]**	**Integral [rel]**	**ν(F2) [ppm]**	**ν(F1) [ppm]**	Integral [abs]	Integral [rel]	ν(F2) [ppm]	ν(F1) [ppm]	Integral [abs]	Integral [rel]	ν(F2) [ppm]	ν(F1) [ppm]
ββ γ1 (left)	565380	0.0133	4.2021	71.6118	786230	0.0123	4.2021	71.6118	278640	0.0091	4.2021	71.6118
ββ γ2 (right)	771290	0.0181	3.8243	71.6557	2591100	0.0178	3.8243	71.6557	553970	0.0181	3.8243	71.6557
β-O-4 α	2397800	0.0564	4.8784	72.2706	1813000	0.0586	4.8784	72.2706	1193500	0.039	4.8784	72.2706
β-O-4 β	1679800	0.0395	4.0815	86.9393	956720	0.041	4.0815	86.9393	936210	0.0306	4.0815	86.9393
β-β α	907350	0.0213	4.6879	85.5779	0.0216	0.0216	4.6879	85.5779	489020	0.016	4.6879	85.5779
Aromatic	42545000	1	7.0056	115.4863	44200000	1	7.0056	115.4863	30625000	1	7.0056	115.4863
β-β β	806140	0.0189	3.0782	54.0005	0.0169	0.0169	3.0782	54.0005	584360	0.0191	3.0782	54.0005
**Negative 1**					**Negative 2**				**Negative 3**			
Object	Integral [abs]	Integral [rel]	ν(F2) [ppm]	ν(F1) [ppm]	Integral [abs]	Integral [rel]	ν(F2) [ppm]	ν(F1) [ppm]	Integral [abs]	Integral [rel]	ν(F2) [ppm]	ν(F1) [ppm]
ββ γ1 (left)	1010700	0.0313	4.2021	71.6118	1923900	0.0292	4.2021	71.6118	1677600	0.0302	4.2021	71.6118
ββ γ2 (right)	1194200	0.037	3.8243	71.6557	2196600	0.0333	3.8243	71.6557	1923000	0.0346	3.8243	71.6557
β-O-4 α	1633200	0.0505	4.8784	72.2706	3612300	0.0547	4.8784	72.2706	2765900	0.0498	4.8784	72.2706
β-O-4 β	1248900	0.0387	4.0815	86.9393	2396700	0.0363	4.0815	86.9393	2008800	0.0361	4.0815	86.9393
β-β α	1221800	0.0378	4.6879	85.5779	2278500	0.0345	4.6879	85.5779	2006700	0.0361	4.6879	85.5779
Aromatic	32309000	1	7.0056	115.4863	65993000	1	7.0056	115.4863	55593000	1	7.0056	115.4863
β-β β	1221100	0.0378	3.0782	54.0005	2286700	0.0347	3.0782	54.0005	2109000	0.0379	3.0782	54.0005

Table S10, NMR Integration values for 24- hour laccase-treated and negative control samples.

This NMR analysis gives rise to their being approximately 0.84 or 1.72 ββ units per 100 C9 units, 4.4 or 4.3 β-O-4 linkages per 100 C9 units in the laccase-treated and negative control after 24 hours.

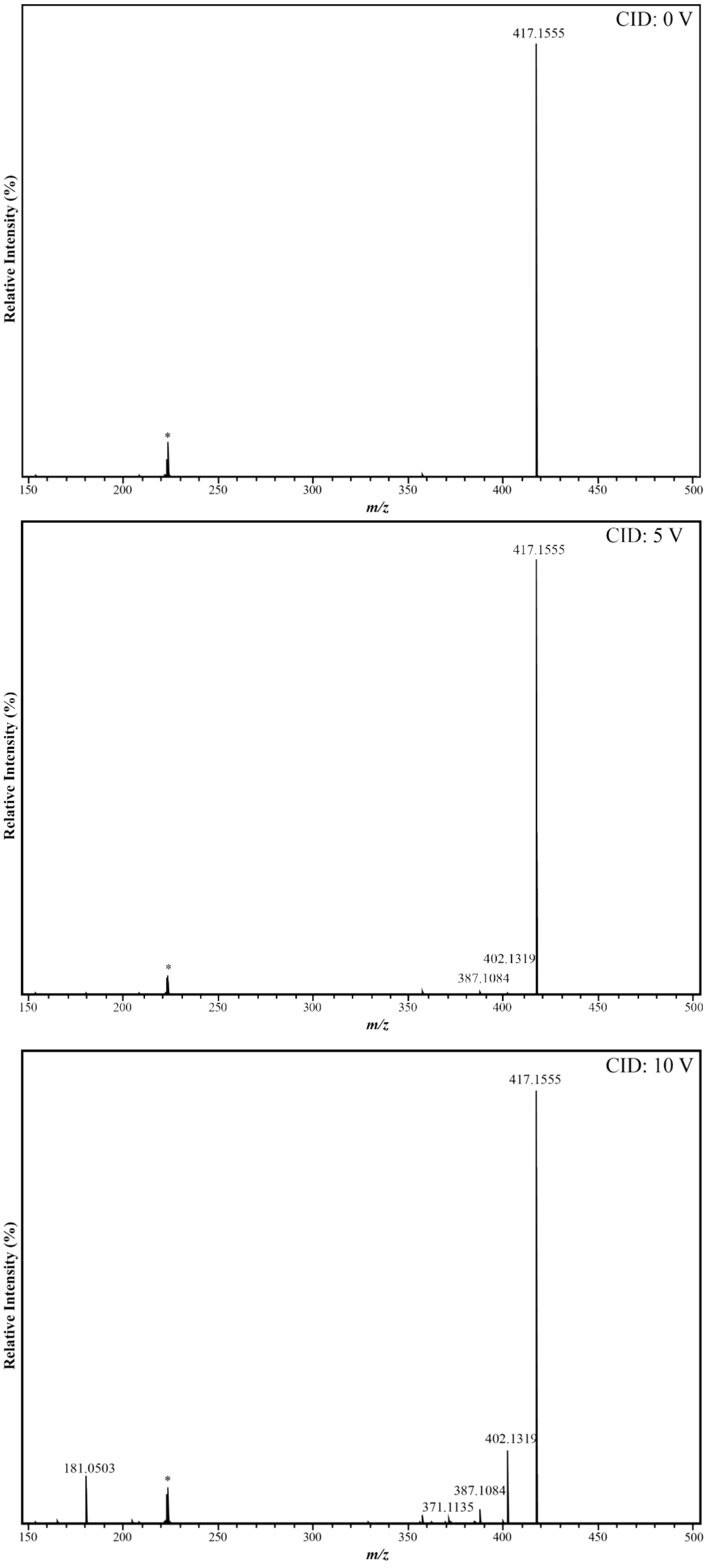

Figure S8. Collision Induced Fragmentation Spectra for the Ion at 417 m/z at 0, 5, and 10 V CID. A contaminating, noise, peak is labelled at 223 m/z is labelled with an asterisk (*). Spectra represent relative intensity where the most abundant peaks is 100% RI.

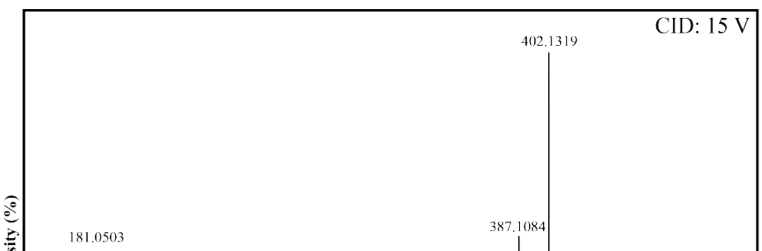

Figure S9. Collision Induced Fragmentation Spectra for the Ion at 417 m/z at 15 and 20 V collision energy, as well as SORI CID. A contaminating, noise, peak is labelled at 223 m/z is labelled with

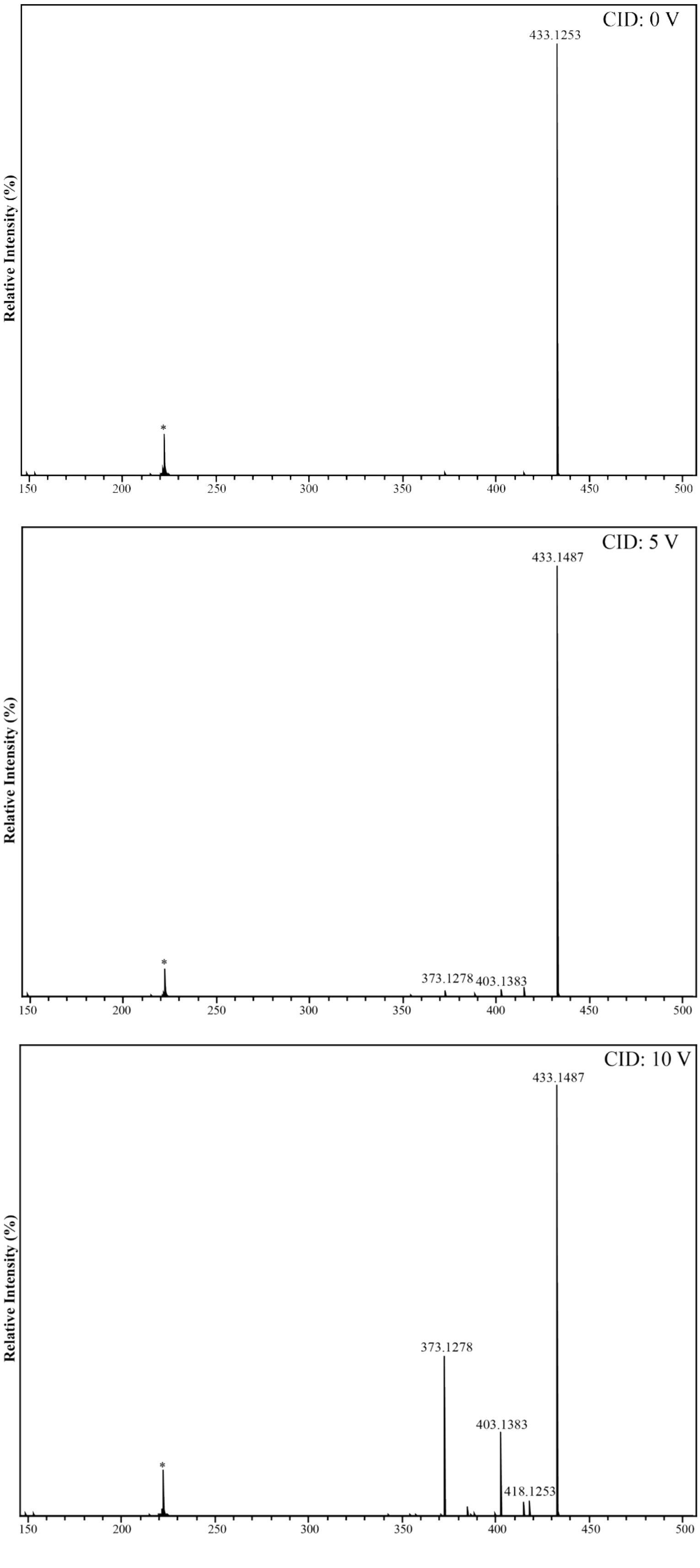

Figure S10. Collision Induced Fragmentation Spectra for the Ion at 433 m/z at 0, 5 and 10 V collision energy. A contaminating, noise, peak is labelled at 223 m/z is labelled with an asterisk (*). Spectra represent relative intensity where the most abundant peaks is 100% RI.

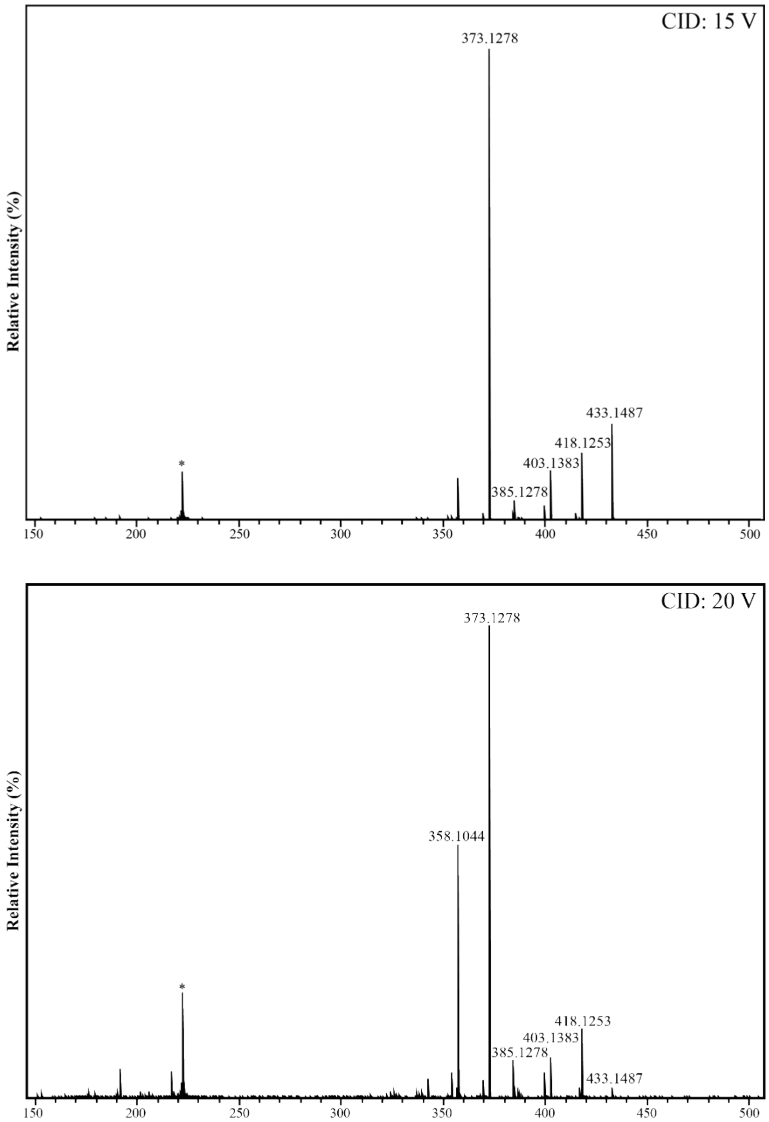

Figure S11. Collision Induced Fragmentation Spectra for the Ion at 433 m/z at 15 and 20 V collision energy. A contaminating, noise, peak is labelled at 223 m/z is labelled with an asterisk (*). Spectra represent relative intensity where the most abundant peaks is 100% RI.

Table S11, Peak List for Ions Present in the CID of the 417 *m/z* Ion at 20 V.

Number	m/z	Intensity
1	166.02679	9887608
2	181.05026	39145764
3	205.08663	1174797
4	315.08714	1588480
5	329.10281	1324511
6	342.11068	1119684
7	354.11071	1149866
8	357.09779	1283046
9	357.13418	2959591
10	371.11349	1093389
11	372.12131	2764836
12	384.12136	2256691
13	387.10845	41126264
14	387.14484	5828986
15	402.12301	1490918
16	402.13197	68225768
17	417.11913	1627753
18	417.1555	36752488

Table S12, Peak List for Ions Present in the CID of the 433 *m/z* Ion at 20 V.

Number	m/z	Intensity
1	28524656	967063
2	5860428	951019
3	7212613	902020
4	26852428	870074
5	5315151	863668
6	325.07146	5362308
7	327.08712	5728000
8	340.09496	6157883
9	343.08206	20621056
10	355.08209	25332272
11	357.09775	9736891
12	358.10558	2.6E+08
13	370.1056	16420440
14	385.09273	36459168
15	385.12912	11446947
16	387.10839	5689142
17	388.11623	5514113
18	400.11625	26720000
19	417.11906	10302458
20	418.12688	72768584
21	433.1504	11976861
